# Unravelling the conundrum of nucleolar NR2F1 localization using antibody-based approaches in vitro and in vivo

**DOI:** 10.1038/s42003-025-07985-1

**Published:** 2025-04-10

**Authors:** Michele Bertacchi, Susanne Theiß, Ayat Ahmed, Michael Eibl, Agnès Loubat, Gwendoline Maharaux, Wanchana Phromkrasae, Krittalak Chakrabandhu, Aylin Camgöz, Marco Antonaci, Christian Patrick Schaaf, Michèle Studer, Magdalena Laugsch

**Affiliations:** 1 Université Côte d’Azur, CNRS, Inserm, Institute of Biology Valrose (iBV), 06108 Nice, France; 2 Institute of Human Genetics, Heidelberg University, Heidelberg, Germany; 3 Hopp Children’s Cancer Center (KITZ), Im Neuenheimer Feld 280, 69120 Heidelberg, Germany; 4 Division of Pediatric Neurooncology, German Cancer Research Center (DKFZ) and German Cancer Consortium (DKTK), Heidelberg, Germany

**Keywords:** Molecular biology, Cell biology

## Abstract

As a transcription factor, NR2F1 regulates spatiotemporal gene expression in the nucleus particularly during development. Aberrant *NR2F1* causes the rare neurodevelopmental disorder Bosch-Boonstra-Schaaf Optic Atrophy Syndrome. In addition, altered *NR2F1* expression is frequently observed in various cancers and is considered a prognostic marker or potential therapeutic target. NR2F1 has been found in both the nucleus and nucleoli, suggesting a non-canonical and direct role in the latter compartment. Hence, we studied this phenomenon employing various in vitro and in vivo models using different antibody-dependent approaches. Examination of seven commonly used anti-NR2F1 antibodies in different human cancer and stem cells as well as in *wild type* and *null* mice revealed that NR2F1 nucleolar localization is artificial and has no functional role. Our subsequent comparative analysis demonstrated which anti-NR2F1 antibody best fits which approach. The data allow for correct data interpretation and underline the need to optimize any antibody-mediated technique.

## Abstract (web summary)

Antibody optimization is crucial for proper data interpretation, such as determining accurate protein localization. Here, this is performed for NR2F1, a transcription factors associated with a rare neurodevelopmental disorder and cancer.

## Introduction

Studying the spatial abundance and role of proteins requires specific antibodies (Abs) to detect and quantify these proteins by widely used methods such as Western blot (WB), flow cytometry (FC) or immunofluorescence (IF). False-positive findings, e.g., due to unspecific staining and false-negative findings, due to the inability to detect an antigen for methodological reasons such as fixation or tissue processing, can have serious implications for data interpretation^1–4^. NR2F1 (COUP-TFI), a transcription factor (TF) of the steroid/thyroid hormone receptor superfamily, is no exception in this regard, as multiple Ab-based experimental approaches have been used in the past to characterize its physiological expression pattern and the pathological consequences of its perturbation. In humans, mutations or deletion of *NR2F1* result in a monogenic neurodevelopmental disorder named Bosch–Boonstra–Schaaf optic atrophy syndrome (BBSOAS)^5–7^. Characterized by intellectual disability, developmental delay, and visual impairment^8,9^, BBSOAS is often associated with additional features, such as epilepsy, autistic traits, hypotonia, oromotor dysfunction, craniofacial malformations, and hearing impairment^10–22^. *NR2F1* is now listed among the 10 top genes causally linked to rare hereditary optic neuropathies^9^. In addition, *NR2F1* has recently been linked to Hirschsprung disease^23^ and Waardenburg syndrome type IV^24^, as well as to the development of lymphoid and cardiac cells^25^, but its precise role in these disorders remains to be investigated.

The NR2F1 protein sequence is highly conserved in evolution^26^ and consists of a DNA-binding domain (DBD), a hinge region (D region), and a ligand-binding domain (LBD) necessary for homo and heterodimerization and co-factor binding^27–29^. Other domains pivotal for NR2F1 function are the activating function (AF) domains 1 and 2^30^, located at the N- and C-terminus, respectively. The high degree of protein sequence conservation, especially between human and mouse NR2F1/Nr2f1 (Nr2f1 carries the same domains, just shifted by a few amino acids), strongly suggests their evolutionarily conserved functions.

As a TF, NR2F1 binds to thousands of targets including genes and their enhancers^31–33^ to regulate developmental processes such as proliferation, migration, and differentiation^34^. In neural development, NR2F1 governs regional identity acquisition of neural progenitors and neurons^27,28,35^ (NP/N) in the developing neocortex^36,37^ and visual system^38,39^, and the balance between excitatory and inhibitory neurons^31,40^. In addition, NR2F1 plays a crucial role in the development of the neural crest, a multipotent and largely embryonic cell population pivotal, for e.g., craniofacial morphogenesis^32,33,41^. Beyond developmental stages, *Nr2f1* expression in the postnatal and adult mouse brains has been linked to neuronal activity^42–44^. NR2F1 also plays an important role in adult non-neuronal tissues, such as peripheral blood^45^, and its aberrant expression has been linked to cancer, serving as a prognostic marker and making it a good candidate for therapeutic development^46–52^.

Both human NR2F1 and mouse Nr2f1 were detected by immunostaining largely within the nucleoplasm^28,38,53–60^. Nevertheless, some previous studies reported a cytoplasmic or nucleolar localization^45,61–63^ raising the possibility that NR2F1 could play an additional non-canonical role in these compartments. Nucleolar aggregates observed after NR2F1 staining of tumor cells with a mouse monoclonal Ab (clone H8132) have been used to quantify NR2F1-positive cells^61–66^, but the specific role of this TF in nucleoli, membraneless organelles formed by phase separation essential for ribosome biogenesis^67,68^, remains unclear.

To explore the possible direct function of NR2F1 in nucleoli, we employed human neural crest cells (hNCC) derived from human induced pluripotent stem cells (hiPSC) and standard IF protocols. By staining with the monoclonal Ab (clone H8132), one of the most commonly used anti-NR2F1 Abs, we were able to confirm the nuclear and nucleolar localization of NR2F1, but the nucleolar-like pattern was also seen in cells that do not express *NR2F1* (e.g., hiPSC). We therefore systematically examined this Ab along with six other NR2F1 Abs using different technical set-ups (IF, FC, WB) in different cancer, *wild type* (*WT*), and CRISPR/Cas9 engineered stem cells, as well as in *WT* and *null* mice. We could not confirm a nucleolar-like pattern of NR2F1 in IF with any of the other Abs, and our results strongly suggest that the nucleolar-like staining by a mouse monoclonal Ab is unspecific and may depend on *NR2F1* expression level, fixation methods, and tissue type.

## Results

### Nucleolar-like localization of endogenous NR2F1 in hNCC observed in IF by the monoclonal Ab (clone H8132)

**Fig. 1:**
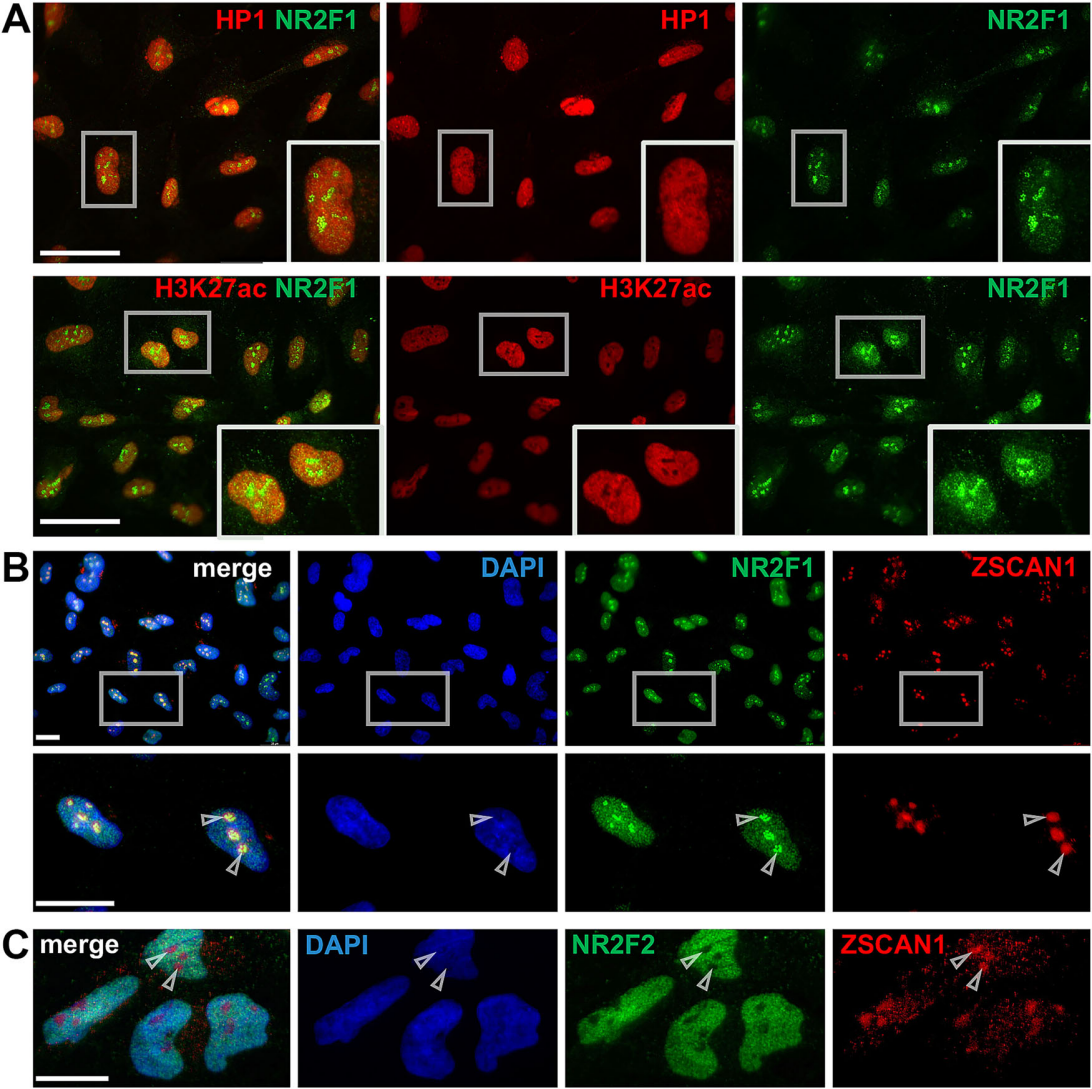
IF of endogenous NR2F1 in hiPSC-derived hNCC using Ab H8132. Gary boxes indicate the selected zoom-in areas. **A** Co-IF of NR2F1 (green) either with HP1 (red) corresponding to inactive chromatin or H3K27ac (red) corresponding to active chromatin status. **B** Co-IF of NR2F1 (green) with ZSCAN1 (red) and their co-localization in nucleoli (gray arrowheads). **C** As indicated by gray arrowheads, co-IF of NR2F2 (green) with ZSCAN1 (red) showed no nucleolar-like localization. Nucleoplasm (blue) was stained with DAPI. Scale bars: 20 µm.

To investigate the expression levels and localization of NR2F1, we used hNCC derived from *WT* hiPSC^33,69,70^. We performed IF using our standard IF protocol^69^ and one of the commonly used NR2F1 monoclonal Ab (clone H8132). After testing three different concentrations (2, 1 µg and 0.4 µg ml^−1^; Supplementary Fig. 1A), we found signals not only diffusely distributed throughout the nucleus but also in aggregate-like clusters, which have previously been observed in hNCC^69^, human malignant cells^62^, prostate^63,71^, and breast cancer^61^ using the same Ab. While the highest Ab concentration resulted in additional staining within the cytoplasm, the lowest recognized mostly the aggregates. Hence, we decided to use for further experiments the intermediate concentration of 1.0 µg ml^−1^, which showed both the diffuse nuclear signal and the nuclear aggregates (Supplementary Fig. 1A). As a negative control, hNCC were stained with either primary Ab H8132 or secondary Ab, anti-mouse Alexa Fluor 488 (AF488), alone, or to capture possible autofluorescence, cells stained only with DAPI to visualize nuclei (Supplementary Fig. 1B). We then tested whether the aggregates stained by the anti-NR2F1 Ab H8132 were specific to NR2F1 or also formed by known NR2F1 co-operators. Cooperative DNA binding of different master TFs at specific loci and their synergistic role in regulating gene transcription in a spatiotemporal manner is crucial for developmental processes and of particular importance for non-coding elements function, such as enhancers, which typically harbor multiple TF binding sites. Consistent with this, chromatin binding sites occupied by NR2F1 in hNCC are frequently co-bound by the hNCC master regulator TFAP2A^32,33^, as shown by chromatin immunoprecipitation coupled with DNA sequencing (ChIP-seq) performed using Ab H8132, which has been widely used for this approach^33,41^ (Supplementary Fig. 1C). Hence, we tested the co-localization of both TFs by co-IF staining (Supplementary Fig. 1D). Although both TFs were diffusely distributed in the nucleoplasm, only NR2F1 and not TFAP2A exhibited the aggregate-like pattern, suggesting that the aggregates may be specific for NR2F1. According to histone modification profiles associated with active (e.g., Histone-3-lysine-27-acetylation, H3K27ac) or repressive chromatin state (e.g., Histone-3-lysine-27-trimethylation, H3K27me3), enhancers (Histone-3-lysine-4-monomethylation, H3K4me1) or promoters (Histone-3-lysine-4-trimethylation, H3K4me3), NR2F1 is associated with active enhancers in hNCC^32,33^ (Supplementary Fig. 1C). Thus, to provide further clues as to what role these NR2F1 aggregates might have, and considering that NR2F1 can activate or inactivate gene expression^26,28,29^, we stained NR2F1 together with H3K27ac, which marks active chromatin, and heterochromatin protein 1 (HP1), which marks an inactive chromatin state^72^. However, none of these co-stainings indicated a convincing co-localization with NR2F1 (Fig. 1A). Since the NR2F1 aggregates were located in DAPI-depleted regions (Supplementary Fig. 1A and D), we hypothesized that they localize to nucleoli. Hence, we performed NR2F1 co-staining with the TF and nucleolar protein ZSCAN1 (zinc finger and SCAN domain containing 1)^73^ and revealed their co-localization (Fig. 1B). In contrast, we found that NR2F2, a homolog of NR2F1, does not colocalize with ZSCAN1 (Fig. 1C), although both TFs share some expression patterns, cooperativity, and target genes^32^. This is consistent with breast cancer data showing that NR2F2 is exclusively localized to the nucleoplasm^74^.

### Evaluation of nucleolar-like localization of NR2F1 with Ab H8132 indicates an unspecific staining

**Fig. 2:**
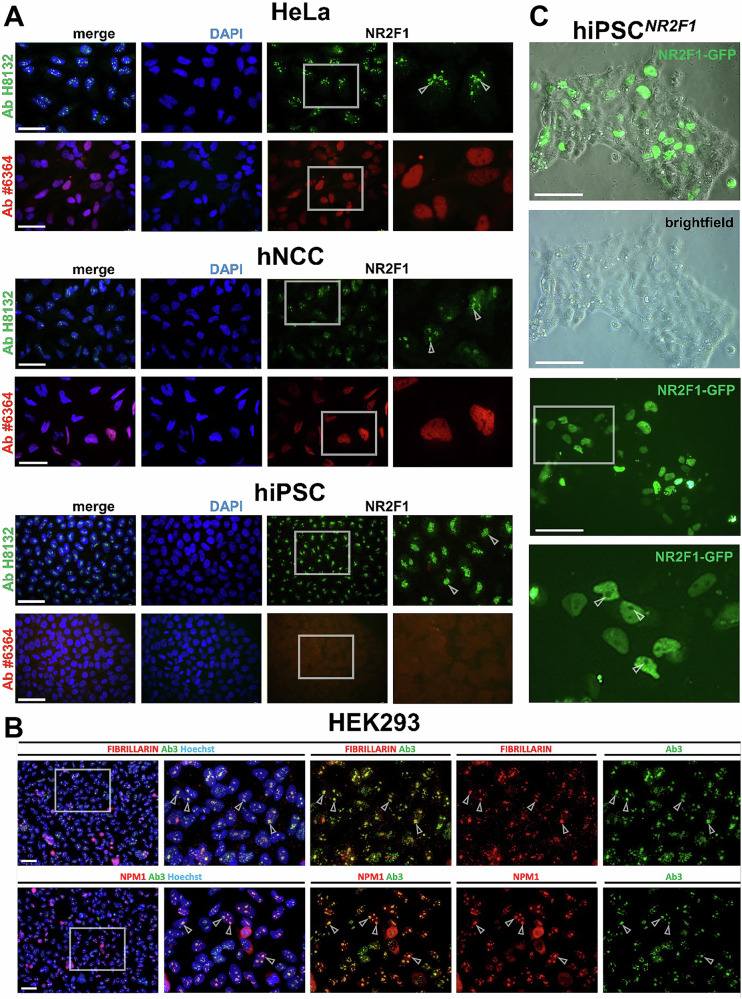
Ab-dependent NR2F1 localization was tested in different cell types. Gray boxes show the selected zoom-in areas. **A** IF of NR2F1 by Ab H8132 (green) or by Ab #6364 (red) was performed in HeLa cells, hiPSC-derived hNCC, and undifferentiated hiPSC. Nucleolar-like foci stained by Ab H8132 are indicated by arrowheads. Ab #6364 stains endogenous NR2F1 only in the nucleoplasm and specifically in HeLa and hNCC, but not in hiPSC, which do not express NR2F1. DAPI marks necleoplasm (blue). **B** IF of NR2F1 Ab H8132 (green), Hoechst (blue), and two different nucleolar markers, Fibrillarin (red) and NPM1 (red). **C** Live-cell imaging of hiPSC^*NR2F1-GFP*^ 24 h after transfection shows nuclear but not nucleolar NR2F1 localization. Zoom-in is marked with a white box and shown for the GFP channel below. Arrowheads point to NR2F1-GFP-negative nucleoli. Scale bars: 50 µm.

Recent studies have shown that a large number of nucleolar proteins are localized to multiple compartments, including the nucleoplasm, and that the nucleolar proteome is much larger than previously recognized^75,76^. To check whether NR2F1 has already been annotated in nucleolar databases, we examined the Human Protein Atlas (ENSG00000175745-NR2F1)^75–77^, Nucleolar Proteome Database (NOPdb)^78^, and UniProtKB/Swiss-Prot (The UniProt Consortium, 2019)^79^, and found no evidence of nucleolar localization of NR2F1. However, these databases lack hNCC-specific data. Because in human prostate cancer samples, the nucleolar localization of NR2F1 was observed in epithelial but not in stromal cells^71^, we hypothesized that the nucleolar localization of NR2F1 might be hNCC-specific. To test this, we used additional cell types for further IF experiments, such as HeLa cells, in which NR2F1 is present in the nucleus (Human Protein Atlas and^80^) but not in nucleoli (HeLa proteomics data^81^). Since NR2F1 is not expressed in undifferentiated pluripotent cells^82^, our undifferentiated hiPSC served as a negative control (Supplementary Fig. 2A). In addition to Ab H8132, we also used another anti-NR2F1 Ab (#6364, Cell Signaling), which targets a different region of NR2F1. Ab H8132 detected the nucleolar-like aggregates in all tested cell types, including the negative control, hiPSC. In contrast, Ab #6364 stained the nucleoplasm only in HeLa and hNCC, but not in hiPSC, and foci were not observed in any of the cells (Fig. 2A). To prove whether the NR2F1 foci in undifferentiated hiPSC by Ab H8132 staining are still localized to nucleoli, we performed a co-staining of NR2F1 or NR2F2 with ZSCAN1. Although, both NR2F1 and NR2F2 (Supplementary Fig. 2A and B) are not expressed in undifferentiated hiPSC, NR2F1, but not NR2F2, showed nucleolar co-localization with ZSCAN1 (Supplementary Fig. 2B). To further confirm the nucleolar nature of the foci, we used two other specific nucleolar markers, Fibrillarin and nucleophosmin (NPM1), co-stained them with NR2F1 Ab H8132 in HEK293 cells and observed clear nucleolar co-localization (Fig. 2B). Since NR2F1 is also not expressed in HEK293 cells, the data provide further experimental evidence that the pattern generated by Ab H8132 is unspecific.

The confounding results of NR2F1 localization prompted us to use a system where we can avoid the use of NR2F1 Abs, such as transiently overexpressing *NR2F1-GFP* (*ENST00000615873.1*) in undifferentiated hiPSC (hiPSC^*NR2F1-GFP*^). We examined the GFP signal in living cells 24 h after transfection (Fig. 2C) and observed a distinct NR2F1-GFP signal only in the nucleoplasm, but not in the nucleoli. This signal, also observed 48 h after transfection differed from the ubiquitous signal resulting from a plasmid expressing GFP without an NR2F1 insert (hiPSC^*GFP*^) (Supplementary Fig. 2D).

To test whether fixation can change the NR2F1-GFP localization and to better distinguish between nucleoplasm and nucleoli, we fixed the hiPSC^*NR2F1-GFP*^ with formaldehyde (FA) for 15 min and stained the cells only with DAPI. Although this slightly diminished the GFP signal^83^, we again detected GFP exclusively in the nucleoplasm but not in nucleoli (Supplementary Fig. 2C first line). In addition, two different anti-GFP Abs (AbGFP) were used (anti-mouse and anti-rabbit), which further confirmed the NR2F1-GFP nucleoplasmic but not nucleolar staining (Supplementary Fig. 2C). We also used hiPSC^*GFP*^ (Supplementary Fig. 2E) and, in contrast to NR2F1-GFP, found ubiquitously expressed GFP.

The assumption that the Ab H8132 signal reflects a technical artifact, was further supported by our bioinformatic analysis of publicly available datasets and algorithms predicting biophysical and functional domains of NR2F1. Nuclear receptors typically contain a highly disordered domain at the N-terminus^84^ and, accordingly, residues 1–81 of NR2F1 are annotated as such (Uniprot ID: P10589), corresponding to the domain detected by Ab H8132. Disordered domains have overlapping biophysical features with low complexity regions (LCRs), which have also been associated with nucleolar proteins, and are typically enriched for specific amino acids like K/E. Using the biophysical tool IUPRED^85^, we found some level of disorder in the N-terminal domain of NR2F1 (Supplementary Fig. 3B) without any K/E enrichment^86–88^ (Supplementary Fig. 3A), and the self-similarity dot plot^87^, revealed only some LCRs for both the short isoform ENST00000615873.1 and the full-length isoform NM_005654 (Supplementary Fig. 3C). This region can therefore be considered as a partial LCR, albeit without the typical sequence composition. Since many nucleolar proteins are themselves direct RNA-binding proteins^89^, we tested this characteristic using DRNApred that—among other factors—takes into account the physical properties of the amino acids, as well as their predicted level of disorder^90^. While an enrichment of DNA-binding residues was predicted in the annotated DBD of NR2F1, no RNA-binding residues were predicted in the protein sequence (Supplementary Fig. 3D). The disordered domain of NR2F1 (1–56 or 1–81, respectively) also shows limited homology with reported nucleolar proteins^76^ according to  BLASTP (Supplementary Fig. 3E). Only eight of the 1308 nucleolar proteins showed local sequence similarity to the NR2F1 N-terminus, and none of these displayed a local sequence identity higher than 61.5%. Although the nucleolar protein ZFHX4 has the highest local sequence identity with NR2F1 1–81 (61.5%), it is unlikely to be bound by Ab H8132 in hiPSC as it is not expressed in human embryonic stem cells (hESC)^32^. Therefore, it cannot explain the nucleolar-like foci detected in hiPSC (Supplementary Fig. 3E). Lastly, we wanted to exclude the possibility that the nucleolar NR2F1 signal was due to indirect NR2F1 binding to a nucleolar protein. As this would not explain the nucleolar NR2F1 signal in cells that lack any NR2F1 expression, we focused on NR2F1-expressing cells, such as hNCC, in which Ab H8132 also stained nucleoli. We therefore manually collected known NR2F1 interactors from four databases (SPRING, IntAct, BioGRID, MINT) and analyzed their overlap with nucleolar proteins (Supplementary Fig. 3E). Five proteins were identified in the overlap, so we next evaluated their co-expression in single-cell RNA-seq (scRNA-seq) data from hiPSC-derived hNCC^69^ (Supplementary Fig. 3F). Three of the five possible NR2F1 interacting proteins with nucleolar localization were not expressed in hNCC (*BCL11B, MPPED2, C1orf210*). *PFDN1* and *ELAVL1* were widely expressed, however, the portion of cells co-expressing *NR2F1* and either of both genes is 66%, with *NR2F1* itself being expressed in only 68% of hNCC. Since we observed a nucleolar signal in virtually all hNCC stained with Ab H8132 (Fig. 1 and Supplementary Fig. 1), the interaction of NR2F1 with any of these nucleolar proteins cannot explain this observation, further supporting the notion that the nucleolar staining cannot be attributed to localization of NR2F1. Thus, our results strongly suggest that the unspecific nucleolar staining in the form of foci caused by IF staining with Ab H8132 may reflect a technical artifact. However, such foci stained with this Ab have not only been reported but also quantified in several studies^61–66^, which may obscure some conclusions. To avoid such complications as much as possible in the future, we systematically tested Ab H8132 and Ab 6364 together with five additional Abs in several commonly used assays and in different cell types.

### All seven Abs are capable of detecting overexpressed NR2F1 in IF, albeit to varying degrees

**Fig. 3:**
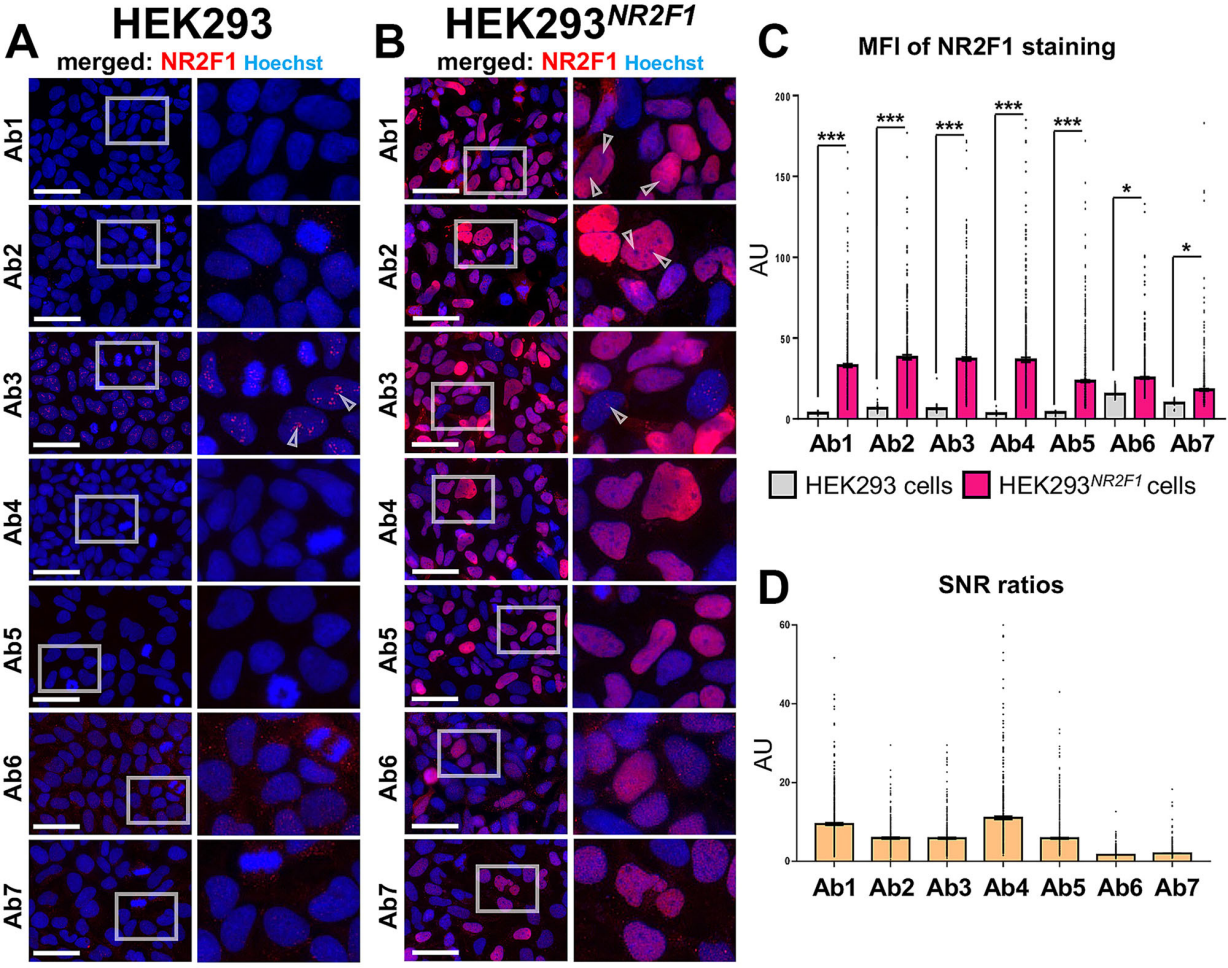
Localization and quantification of NR2F1 levels by IF in untransfected HEK293 and HEK293^*NR2F1*^ cells. **A** and **B** Gray boxes in the first column show the selected zoom-in area placed next to it. Nucleoplasm (blue) was stained with Hoechst. Scale bars: 50 µm. **A** Negative control: untransfected HEK293 cells that do not express NR2F1 endogenously. Arrowheads indicate nucleolar-like foci exclusively in the Ab3 staining. **B** HEK293^*NR2F1*^ cells 48 h after transfection with an NR2F1 plasmid. Arrowheads indicate examples of nucleoli typically not stained by Ab1 and Ab2 and nucleolar-like foci stained by Ab3, seen only in cells that were unlikely to have been successfully transfected. **C** MFI of histograms resulting from NR2F1 staining measured for each Ab used (x-axis) in untransfected control HEK293 cells (gray columns) and in HEK293^*NR2F1*^ cells (pink columns). The data are shown in arbitrary units (AU) and represented as mean ± SEM; the MFI was measured in at least 330 and up to 800 cells per sample. **D** SNRs between the MFI of HEK293^*NR2F1*^ and untransfected HEK293 cells are shown in AU as mean ± SEM for each Ab.

To clarify the localization of NR2F1, a total of seven anti-NR2F1 Abs (Ab1–Ab7) were used, including Ab H8132 (hereafter referred to as Ab3) and Ab 6364 (hereafter referred to as Ab5) (Supplementary Fig. 4A). The distinct regions of the NR2F1 protein that have been used as immunogens to generate these Abs, are schematized in Supplementary Fig. 4A, and the main additional information extracted from available data sheets is summarized in Supplementary Fig. 4B. To test these Abs, we transfected HEK293 cells with the commercially available NM_005654 NR2F1 isoform, which was used as an immunogen. However, this information is not available for all Abs. Untransfected and NR2F1-overexpressing HEK293 cells (HEK293^*NR2F1*^) were fixed with paraformaldehyde (4% PFA for 15 min) 48 h after transfection (Fig. 3). In untransfected cells, all Abs generally showed no or weak background signals (Ab6 and Ab7 weak cytoplasmic background, Fig. 3A last two lines). The nucleolar-like foci were observed exclusively in the Ab3 staining (Fig. 3A, third row with empty arrowheads), which is consistent with our previous results from hiPSC, hiPSC-derived hNCC, and HeLa cells (Figs. 1 and 2). However, in HEK293 cells, the foci were much weaker, which might be related to the morphological diversity (e.g., size and number) and/or composition of nucleoli across different cells and conditions, such as cell cycle or stemness^91,92^. Overall, all Abs were able to detect overexpressed NR2F1 in the nucleus, but with different intensities (Fig. 3B). Ab5, 6, and 7 detected NR2F1, although they were not recommended for IF according to the manufacturer (Supplementary Fig. 4B). To better quantify the specificity and sensitivity of the NR2F1 Ab panel, we calculated the mean fluorescence intensities (MFI), and signal-to-noise ratios (SNRs, intensity signal in transfected cells versus background in untransfected cells) of all Abs (Fig. 3C, D). However, it should be noted that we used our standard IF protocol for all Abs, and did not optimize specific parameters for each individual Ab, such as incubation time. Nevertheless, our data show that Ab1-5 had the best SNRs, while Ab6 and Ab7 had a rather poor ratio (Fig. 3D). However, regardless of the SNR, Ab3 was the only Ab that additionally stained the nucleolar-like foci, but these were visible in only a few cells, apparently only those that had not been successfully transfected with NR2F1 (Fig. 3B indicated by arrowheads). Since our data strongly suggest that the unspecific nucleolar-like staining by Ab3 inversely depends on the NR2F1 protein levels, we can speculate that a lower affinity and most likely unspecific target is only detected if Ab3 cannot bind to the higher affinity target NR2F1.

To corroborate the unspecific staining of NR2F1 by Ab3, we co-stained Ab3 and Ab1 as well as Ab2 and Ab3 in untransfected (Supplementary Fig. 5A) and HEK293^*NR2F1*^ cells (Supplementary Fig. 5B). Moreover, to exclude the possibility of any background signal being enhanced by the secondary Ab, we used two secondary Abs conjugated to either AlexaFlour 555 (AF555) or 647 (AF647) in two different combinations (Supplementary Fig. 5A and B). Once again, only Ab3 stained nucleoli-like foci in untransfected cells, regardless of which secondary Ab was used, while Ab2 and Ab1 showed no significant background or unspecific staining (Supplementary Fig. 5A). In HEK293^*NR2F1*^, the NR2F1 staining resulting from Ab3 co-localized with Ab1 and Ab2 within the nucleus (Supplementary Fig. 5B). In a few cases, nucleolar-like foci were observed only for Ab3, where the cells were not co-stained with Ab1 or Ab2, and therefore only in cells that were not successfully transfected with NR2F1 (arrowhead in Supplementary Fig. 5B). To further prove that the nucleolar-like foci resulting from Ab3 staining are unspecific, we stained with either Ab3 or Ab1 together with an AbGFP in hiPSC^*NR2F1-GFP*^ (Supplementary Fig. 5C and D). Double-positive cells for AbGFP and Ab3 staining (regardless of AbGFP or GFP signals) showed signals exclusively in the nucleoplasm, and nucleolar-like foci were visible only in GFP-negative hiPSC (Supplementary Fig. 5C). In contrast, all double-positive cells for GFP and Ab1 co-localized exclusively in the nucleoplasm of successfully transfected hiPSC, with Ab1 signal being absent in GFP-negative cells (Supplementary Fig. 5D). To further confirm the unspecific nature of the nucleolar staining visible when using Ab3, we also stained hiPSC^*GFP*^ with Ab1 and Ab3. While Ab1 did not show any signals in these cells, Ab3 resulted in a strong pattern in the nucleoli of both successfully transfected (hiPSC^*GFP*^ positive) and untransfected (hiPSC^*GFP*^ negative) cells (Supplementary Fig. 5E). Thus, our data provide further evidence that the nucleolar foci appear only when using Ab3, are unspecific, and particularly obvious when NR2F1 expression is below a certain level that remains to be determined.

### Only four out of seven Abs are able to detect endogenous NR2F1 in hiPSC-derived NP/N, although to different degrees

**Fig. 4:**
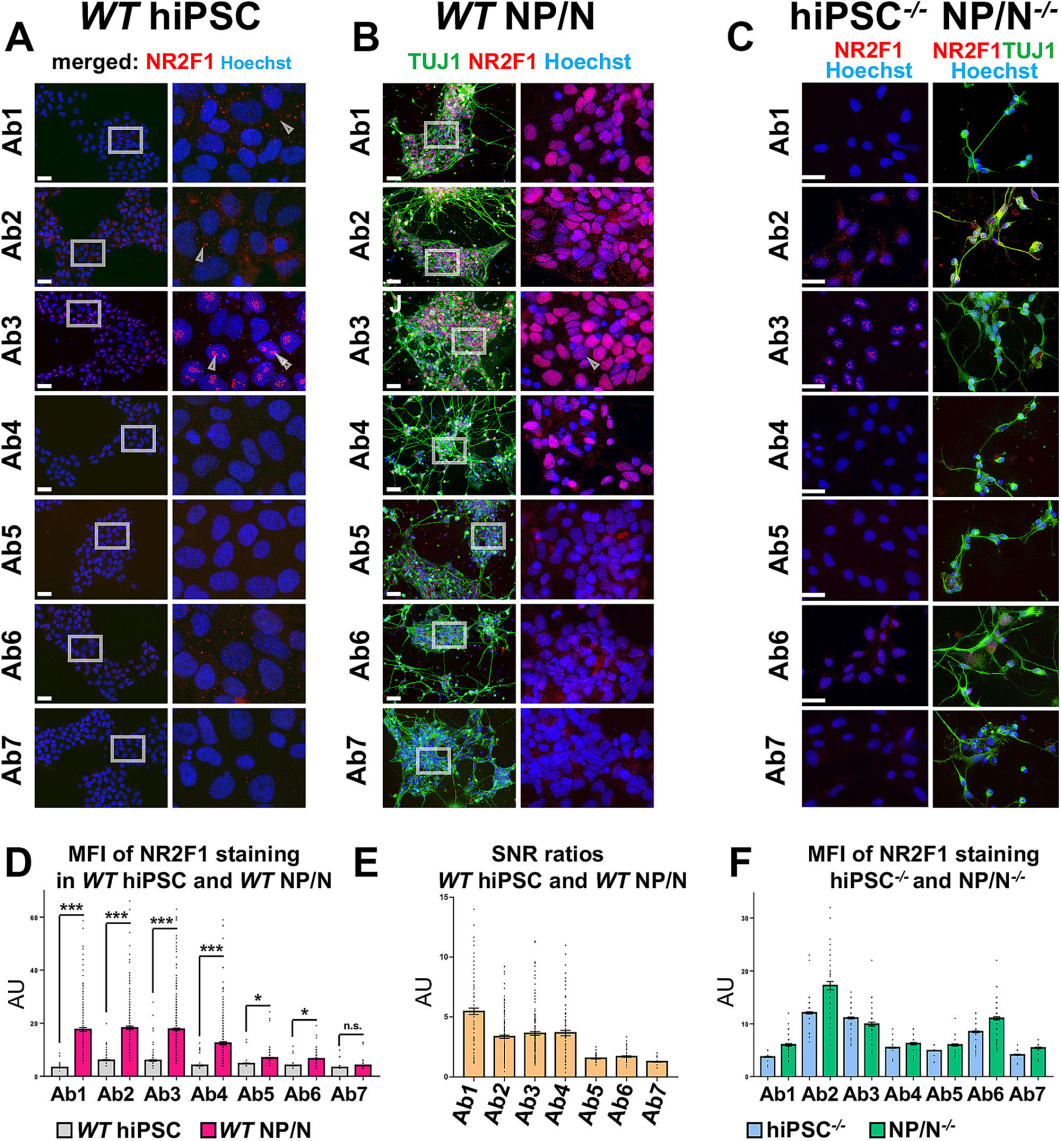
Detection of endogenous NR2F1 in *WT* and hiPSC^*−/−*^, NP/N^*−/−*^ by IF using seven Abs. **A** and **B** Gray boxes show the selected zoom-in area placed next to it. (**A**-**C**) The neuronal marker TUJ1 (green) was used only for IF in differentiated cells. Nucleoplasm (blue) was stained with Hoechst. Scale bars: 50 µm. The corresponding zoom-in is next to each image. **A** Undifferentiated *WT* hiPSC: arrowheads point to unspecific IF signals in the cytoplasm after Ab1 and Ab2 staining and to nucleolar-like foci after Ab3 staining. **B** NP/N derived from *WT* hiPSC. **C** Undifferentiated hiPSC^*−/−*^ with arrowheads indicating nucleolar-like foci stained by Ab3 as well as NP/N^*−/−*^, which showed no foci. **D** MFI of each NR2F1 staining (Abs1–7) in AU (mean ± SEM) measured in the nucleus of undifferentiated hiPSC (gray columns) and of hiPSC-derived NP/N (magenta columns) in at least 120 cells per sample (up to 500 cells per sample). **E** SNRs measured for each Ab (Abs1–7) in undifferentiated *WT* hiPSC and hiPSC-derived *WT* NP/N shown in AU as mean ± SEM. **F** MFI measured at least in 60 and up to 150 cells per sample of each NR2F1 staining (Ab1–Ab7) displayed as mean ± SEM in undifferentiated hiPSC^*−/−*^ (blue columns) and NP/N^*−/−*^ (green columns).

Next, we systematically tested all seven Abs in IF using *WT* hiPSC-derived NP/N endogenously expressing NR2F1^93,94^ in comparison to undifferentiated hiPSC that do not express NR2F1 (Figs. 2A, 4A, B, and Supplementary Fig. 2A, B). While Ab1, 2, 3, and 6 gave rise to unspecific signals in undifferentiated hiPSC, but to varying degrees and mostly in the cytoplasm, only Ab3 displayed pronounced nucleolar-like foci (Figs. 2A, 4A and Supplementary Fig. 2B). In addition to NR2F1, NP/N were co-stained with TUJ1 to confirm their neuronal identity (Fig. 4B and C). Overall, Ab5, 6, and 7 failed to detect endogenous NR2F1, and only Ab1, 2, 3, and 4 successfully stained endogenous NR2F1 in the nucleus, but to varying degrees (Fig. 4B). We noticed that in some cells, endogenous NR2F1 was not stained or only faintly stained (Fig. 4B), presumably due to the heterogeneity of the differentiated cells^95^. In contrast to hNCC (Fig. 1), Ab3 only occasionally stained few and small nucleolar-like foci in our NP/N (Fig. 4B). These data are consistent with our previous results (Figs. 2 and 3) indicating that this artifact depends on NR2F1 expression levels and nucleoli dynamics, which vary between cell types^96,97^. The MFI values and SNR indicate that Abs1-4 detect endogenous NR2F1 well (Fig. 4D and E) but for Ab3, this calculation may not be entirely reliable due to the nucleolar-like staining. Of note, among the Abs5-7 that barely or not at all detected endogenous NR2F1 in *WT* hiPSC-derived NP/N, Ab5 and Ab7 are not recommended by the manufacturer for IF, whereas Ab6 is (Fig. 3B).

To further validate our results, we used CRISPR-Cas9-engineered hiPSC with a homozygous indel in exon 2 (of ENST00000615873.2 or exon 1 of NM_005654.6, with gRNA targeting hg38, chr5:93,585,331) that results in a premature stop codon (hiPSC^*−/−*^). We then differentiated hiPSC^*−/−*^ into NP/N^−/−^, confirmed their neuronal identity by TUJ1 staining (Fig. 4C) and, together with undifferentiated hiPSC^*−/−*^ (Fig. 4C, first column) stained them with the seven NR2F1 Abs. In both undifferentiated hiPSC^−/−^ and differentiated NP/N^−/−^, Ab2, 3, and 6 showed unspecific NR2F1 staining as indicated by the MFI (Fig. 4F). Since Ab3 still shows nucleolar-like foci in hiPSC^*−/−*^, this localization can now confidently be considered unspecific (Fig. 4C, first column).

### IF of endogenous Nr2f1 in mouse embryonic brain sections

**Fig. 5:**
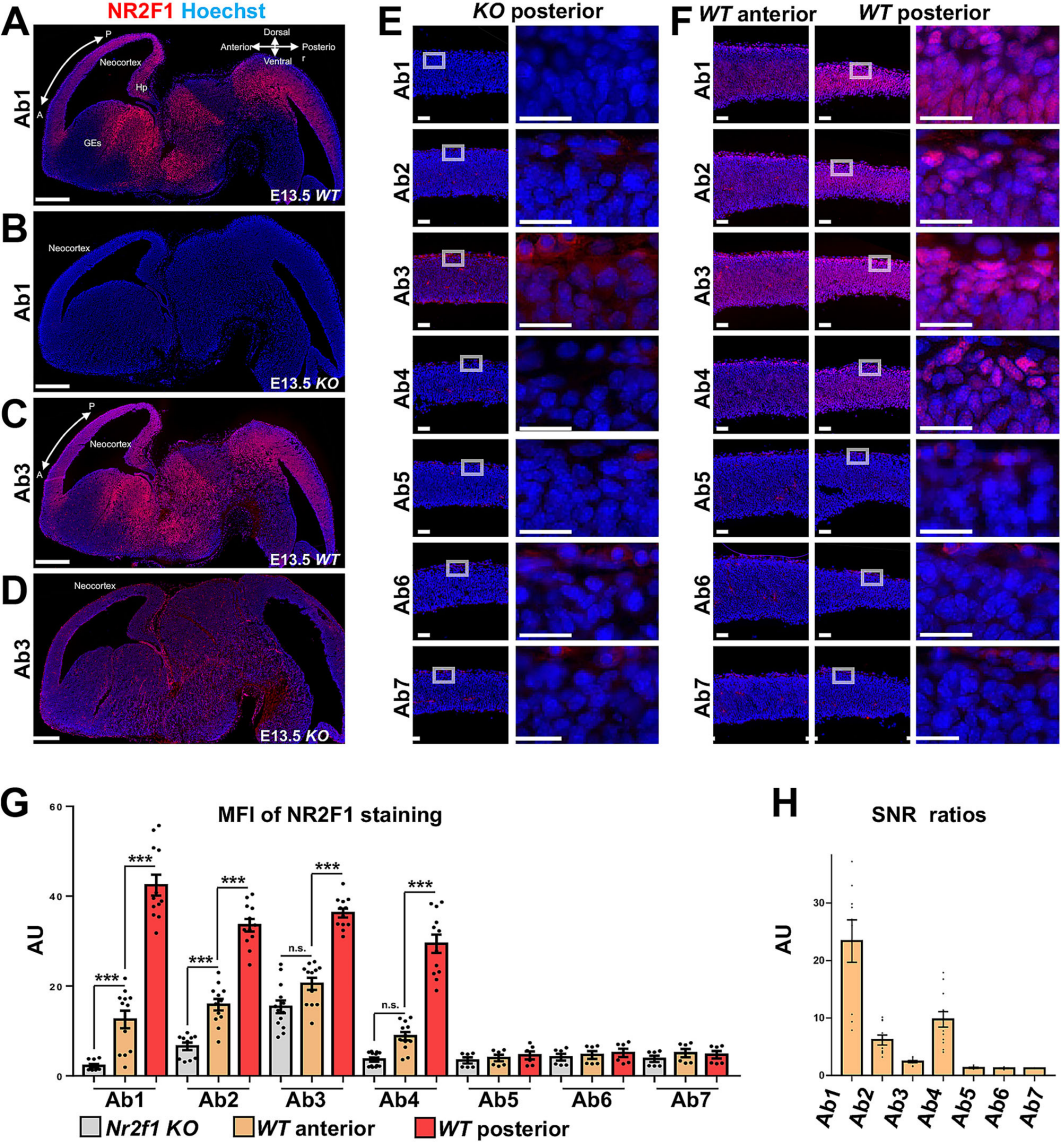
Comparison of the anterior-posterior Nr2f1 gradient in *WT* and *KO* mouse brains at E13.5 in IF using seven Abs. **A**–**F** Sagittal sections of whole *WT* and *KO* mouse brains. Nucleoplasm (blue) was stained with Hoechst. **A**–**D** The overview of IF staining with Ab1 and Ab3 in *WT* and *KO* shows a strong background signal in the case of Ab3 in *KO* with a schematic of the anterior–posterior and dorsoventral axes shown in (**A**). Scale bars: 500 µm. **E** Posterior images of *Nr2f1 KO*. Greay boxes indicate the area of zoom-in shown next to it. **F** Anterior areas and posterior areas in *WT*. Gray boxes in **E** and **F** indicate zoom-in areas. Only Abs1-4 can detect Nr2f1, but Ab3 leads to high background staining localized in the cytoplasm or extracellular matrix rather than in the nucleoli. Scale bars: 50 µm in the first column of **E** and first and second columns of **F**; 25 µm in the last column of (**E**) and (**F**). **G** MFI of each Nr2f1 staining measured in random fields from at least 6 and up to 12 different cortical regions per sample in *Nr2f1 KO* and *WT* brains represented as mean ± SEM in AU. **H** SNRs (*WT* vs. *KO*).

In the next step, we decided to systematically test all seven Abs in cryostat-sliced samples of mouse embryonic brain at E13.5 (Fig. 5). It has been previously shown that Nr2f1 is expressed in the neocortex of the mouse brain in a gradient from anterior-low to posterior-high, which is essential for the formation of different cortical identities^36,98,99,101^. Hence, we compared Nr2f1 levels along the anterior-posterior axis of the developing mouse brain in *WT* and *null* mice, with the latter lacking any Nr2f1 protein expression^55^ (referred to as *KO*). All cryostat-sliced brain samples were subjected to a minimum of 2 h PFA fixation, which is a standard procedure for staining mouse tissue, followed by cryoprotection in sucrose and freezing^34,100^. Figure 5A–D shows whole brain sections at E13.5 of *WT* (Fig. 5A and C) and KO mice (Fig. 5B and D) stained with either Ab1 (Fig. 5A and B) or Ab3 (Fig. 5C and D). In agreement with previous studies^28,36,101^, we observed an Nr2f1 gradient along the anterior-posterior axis of the dorsal telencephalon in the *WT* mouse, when using Ab1 or Ab3 (Fig. 5A and C). In the *KO* mouse, the absence of Nr2f1 was confirmed by Ab1, but an unspecific background in the cytoplasm or extracellular matrix rather than nucleoli was observed for Ab3 (Fig. 5B and D). To get a more accurate picture of Nr2f1 localization, we examined different cortical regions of *WT* and *KO* mice at a higher magnification for all seven Abs (Fig. 5E and F). In total, only Abs1–4 were able to detect Nr2f1, but comparing *WT* anterior to posterior Nr2f1 levels, Ab1, Ab2, and Ab4 displayed the clearest gradient (Fig. 5E and F). Measuring MFI and calculation of SNRs further confirmed our observations (Fig. 5G). The data suggest that the anterior–posterior Nr2f1 gradient (Fig. 5C, G, and H) may be masked in the Ab3 staining of the *WT* brain sections by the high background. Consistent with our previous findings, the results further suggest that the nucleolar epitope to which Ab3 binds unspecifically may be cell-type specific. Besides this, the unspecific staining observed with Ab3 in the cytoplasm or extracellular matrix of the mouse brain (Fig. 5) could also be caused by the different staining protocols resulting from mouse tissue processing, suggesting that the cell type and/or fixation method could have a major impact on the presence of artefactual nucleolar-like staining when using Ab3.

### The nucleolar-like foci observed in IF by Ab3 depend on fixation time and type

To test our hypothesis that the presence or absence of nucleolar-like foci appearing after staining with Ab3 might depend on the fixation time and/or method, we modulated these parameters. To this end, we used HeLa cells expressing endogenous NR2F1 and compared Ab3 staining after three different fixation methods: standard cross-linking with 3.7% FA, dehydration fixation with either acetone/methanol mix or ethanol with glacial acetic acid (GA) and different fixation times (5, 10, 30 and 60 min). We also pre-treated the cells for 30 s with or without 0.5% Triton X-100 to pre-clear the nuclei (Supplementary Fig. 6A–F). Longer duration (60 min) of standard cross-linking with 3.7% FA with or without Triton pre-treatment reduced not only the unspecific nucleolar-like but also the nucleoplasmic NR2F1 staining (Supplementary Fig. 6A and D). In contrast, both methods of dehydration fixation without Triton pre-treatment resulted in strong nuclear and unspecific cytoplasmic staining (Supplementary Fig. 6B and C). Remarkably, Triton pre-treatment followed by 60 min dehydration fixation with acetone/methanol resulted in nucleoplasmic rather than focal nucleolar-like staining (Supplementary Fig. 6E–H). A similar tendency was observed after pre-treatment with Triton for 30 s and 60 min of dehydration fixation with ethanol/GA (Supplementary Fig. 6F). Overall, our results suggest that the nucleolar-like foci stained by Ab3 can be reduced in HeLa cells endogenously expressing NR2F1 by increasing the fixation time and performing dehydration fixation, instead of cross-linking fixation. (Supplementary Fig. 6). These data are consistent with our results from mouse brains after IF using Ab3, in which prolonged fixation and dehydration resulted in the loss of nucleolar foci, but also an increase in unspecific staining of the extracellular matrix (Fig. 5).

**Fig. 6:**
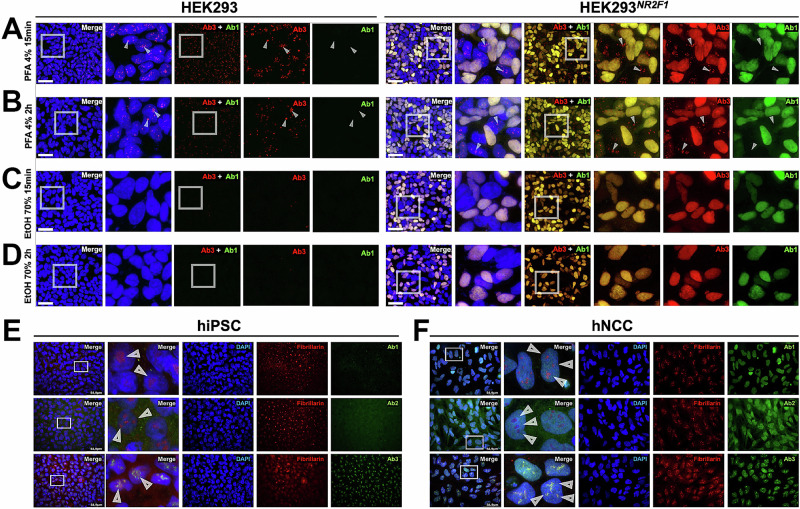
Different fixation times and conditions for IF staining with Ab3 and nucleolar co-localization of signals resulting from Ab3 with fibrillarin. Gray boxes show the selected zoom-in area placed next to it; arrowheads indicate nucleolar areas. **A**–**D** Co-staining of NR2F1 with Ab1 (green) and Ab3 (red) in untransfected HEK293 cells and 48 h post-transfection in HEK293^*NR2F1*^ cells. Nucleoplasmi was stained by Hoechst. Only successfully transfected HEK293^*NR2F1*^ cells display double-positive nuclei co-stained by Ab1 and Ab3. **A** PFA fixation for 15 min and **B** 2 h reduced the unspecific nucleolar-like foci resulting from Ab3 staining without affecting the Ab1 signal. **C** 15 min and **D** 2 h fixation with 70% ethanol completely prevented the occurrence of unspecific nucleolar-like foci in the Ab3 staining without affecting the Ab1 staining. Scale bars: 50 µm. **E** and **F** Scale bars: 59 µm. Co-staining of fibrillarin (red) with Ab1, Ab2, and Ab3 (red). **E**–**F** Nucleoplasm was stained by DAPI (blue). **E** *WT* hPSC shows a specific nucleolar pattern stained by Ab3 that overlaps with fibrillarin in DAPI-depleted regions. **F** In *WT* hNCC cells, all Abs stain the NR2F1 nucleus (DAPI-positive but fibrillarin-negative regions), but only Ab3 overlaps with fibrillarin in DAPI-depleted regions.

Since we previously showed that the background signal and nucleolar-like appearance in IF, when staining with Ab3, also depends on the NR2F1 expression level (Fig. 4, Supplementary Fig. 5), we next tested how the fixation protocol can influence Ab3 NR2F1 staining in our HEK293^*NR2F1*^ cells (Fig. 3). The advantage of this system is that, after transfection, we obtained a population of cells with different NR2F1 expression levels. The transfected cells with high NR2F1 expression can be distinguished from the non-transfected cells that do not express NR2F1 and serve as an internal negative control. Thus, we fixed HEK293^*NR2F1*^ cells 48 h after *NR2F1* transfection with two different fixatives for different times, prior to staining with Ab1 and Ab3. Our standard 15-min PFA fixation served as a control (Fig. 6A and B). In untransfected cells, the unspecific nucleolar-like foci seen for the staining with Ab3 became partially weaker after 2 h fixation with PFA, whereas in successfully transfected HEK293^*NR2F1*^ cells, nuclear NR2F1 co-stained with Ab1 and Ab3 was observed (Fig. 6A and B). Therefore, we concluded that in HEK293 cells, a PFA fixation prolonged to 2 h instead of 15 min leads to a slight loss of the nucleolar-like foci staining, without increasing the unspecific cytosolic or extracellular signal (Fig. 5). Finally, we fixed the untransfected and HEK293^*NR2F1*^ cells with ethanol for 15 min or 2 h and stained them with Ab1 and Ab3 and observed a total absence of the nucleolar-like signal in untransfected cells and only the nucleoplasmic localization of NR2F1 in transfected cells (Fig. 6C and D). To ensure an accurate interpretation, we co-stained Ab3 with the nucleolar marker fibrillarin, which further confirms previous findings (Supplementary Fig. 6I). We also stained fibrillarin together with Ab1, Ab2, or Ab3 in hiPSC that does not express NR2F1 and in hNCC expressing NR2F1. As shown in Fig. 6E, Ab1 and Ab2 do not cause signals in hiPSC, while Ab3 stains foci, which clearly colocalize with fibrillarin. In hNCC all three Abs stain NR2F1 in the nucleus, but only Ab3 causes additional staining of nucleolar foci that overlap with fibrillarin (Fig. 6F). These results support our hypothesis that NR2F1 is not expressed in the nucleoli and allowed us to determine 70% ethanol as the optimal fixation method for Ab3 to obtain valid NR2F1 IF staining. Thus, we next systematically tested the whole panel of seven NR2F1 Abs with other Ab-mediated techniques.

### Comparative analyses of seven anti-NR2F1 antibodies revealed which of them best fit for FC and WB

**Fig. 7:**
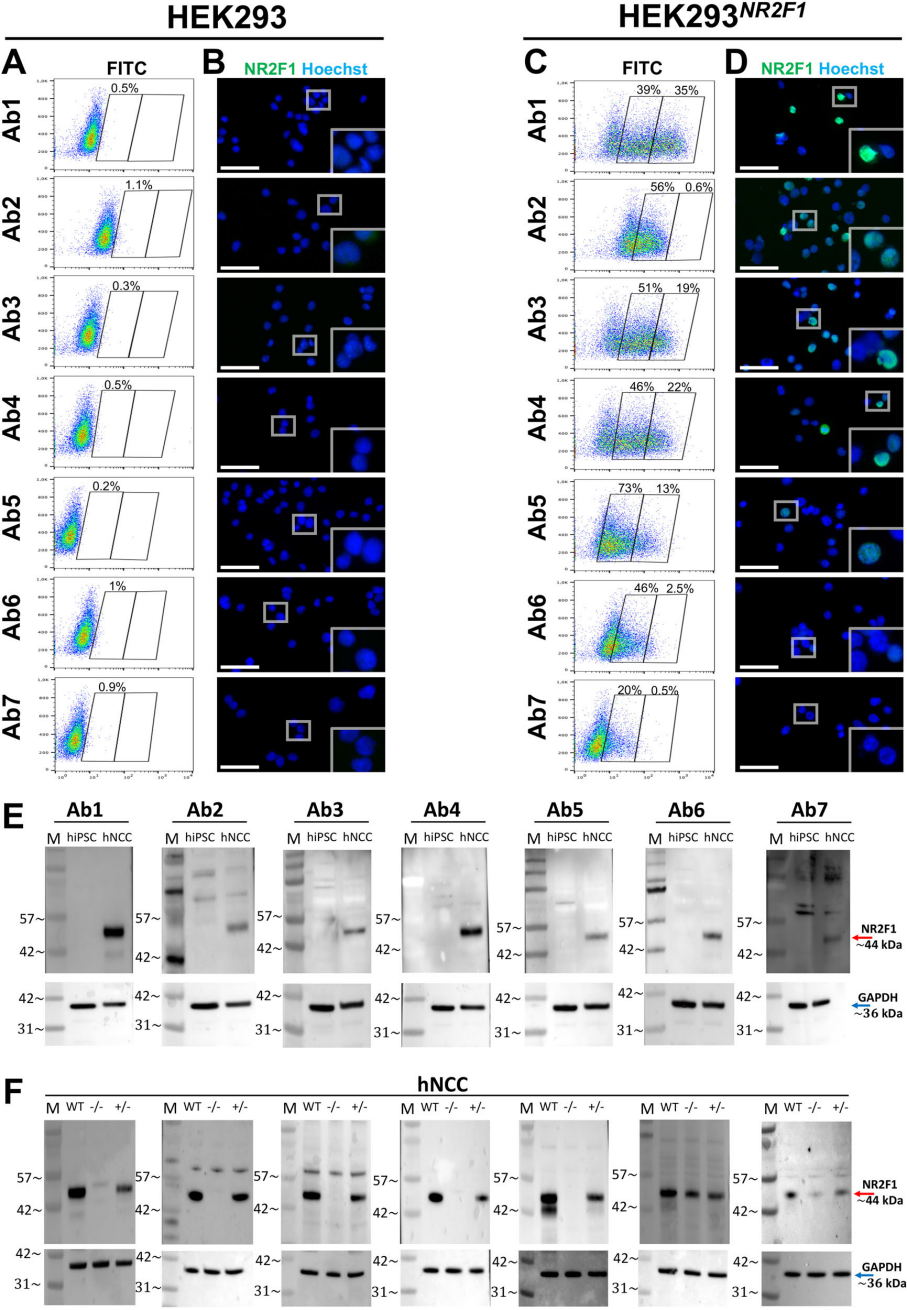
Testing all of the seven anti-NR2F1 Abs in FC and WB. **A**–**D** Quantification of NR2F1 levels by FC using all seven Abs in HEK293 and HEK293^*NR2F1*^ cells. **A** and **B** Untransfected and **C** and **D** HEK293^*NR2F1*^ cells were fixed with 70% ethanol 48 h after transfection, and stained with the primary Abs, followed by adequate AF488 as a secondary Ab. **A** and **C** FC data. For each sample, two batches with each 10,000 events were measured using the same settings and show that Ab1, Ab3, and Ab4 detect NR2F1 best under these conditions. **B** and **D** After FC measurement, the cell suspensions of the remaining samples were stained with Hoechst (blue), spotted on glass slides, and visualized by fluorescence microscopy. Scale bars: 50 µm. **E** and **F** Detection of NR2F1 in WB in samples from *WT* hiPSC and hNCC as well as hNCC^*−/−*^ and hNCC^*+/−*^. 20 µg per sample of RIPA lysate was loaded. Red arrows mark the 44–46 kDa band corresponding to the specific size of NR2F1, blue arrows mark GAPDH (~36 kDa) used as loading control. **E** Undifferentiated *WT* hiPSC and hNCC. **F** hNCC^*−/−*^ and hNCC^*+/−*^. All Abs recognized the 44–46 kDa band corresponding to NR2F1 hNCC^*+/−*^, and, depending on the Ab, several other bands. Ab6 and Ab7 also recognized the 44–46 kDa band in hNCC^*−/−*^, which was not detected by other Abs. M molecular size marker.

Since Ab3 staining accuracy was dependent on the level of NR2F1 expression (Figs. 2, 3, Supplementary Figs. 2C and 4), we used FC to quantify NR2F1 protein expression at the single cell level. To obtain a population of cells with different NR2F1 expression levels, we utilized HEK293^*NR2F1*^ cells (Figs. 3 and 6) and assessed the ability of all seven Abs to detect intracellular NR2F1 after fixation and permeabilization with 70% ethanol. Using this system, we could not only determine NR2F1-negative and -positive (NR2F1^total^) cells but also distinguish between low (NR2F1^low^) and high (NR2F1^high^) positive cells (Fig. 7A–D, Supplementary Fig. 7). Because each Ab staining was derived from the same pool of HEK293^*NR2F1*^ cells harvested 48 h after transfection, we can assume that the same number of transfected cells were present in each sample being compared. All samples were measured at the same voltage, which directly corresponds to the fluorescence intensity of the highly variable background of each Ab staining in the untransfected cells (Fig. 7A). Overall, Ab1, Ab3, Ab4, and Ab5 showed the highest percentage of NR2F1^total^ cells (68.5–86.1%), whereby only 13.6–34.7% of the cells exhibited NR2F1^high^ levels. Although Ab5 displayed the highest number of NR2F1^total^ cells (86.1%), this corresponded mainly to NR2F1^low^ cells (72.5%), suggesting a limited ability of this Ab to detect a genuine NR2F1^high^ level upon overexpression. While Ab2 and Ab6 gave rise to only NR2F1^low^ cells (45.5–55.7%), Ab2 showed by far the lowest NR2F1^low^ signal, namely 0.54% (Fig. 7C). After FC, the remaining unstained and stained cells were then plated on glass for direct visualization with a fluorescence microscope (Fig. 7B, D). Although the staining intensity measured by FC and microscopy cannot be directly compared^102^, the microscopy images showed no nucleolar-like pattern of NR2F1 after staining with Ab3. This is consistent with the previous optimization of the fixation method (Fig. 6A–D). Thus, our FC data show that Ab1, 3 and 4 are most suitable to measure NR2F1 expression in HEK293^*NR2F1*^ cells after ethanol fixation, and these Abs can be used to precisely quantify different degrees of NR2F1 expression degree at single cell level (Fig. 7A–D, Supplementary Fig. 7). However, as only Ab1 is recommended for FC (Supplementary Fig. 4B), NR2F1 staining with Ab2, 5, 6 and 7 for this purpose may require further optimization.

In contrast to IF and FC, standard WB separates denatured proteins, by molecular mass, allowing a general evaluation of Ab specificity. Hence, we systematically tested all seven Abs in WB, using *WT* hiPSC-derived hNCC that endogenously express NR2F1^32,33,41^ and undifferentiated hiPSC that do not express it^82^ (Supplementary Fig. 2A). Since all Abs were able to detect a clear 44–46 kDa band only present in hiPSC-derived hNCC but not in undifferentiated hiPSC (Fig. 7E), we assume that this band corresponds to the NR2F1 protein. However, we cannot distinguish conclusively between the *ENST00000615873.1* isoform (44 kDa, previously determined in our HeLa, hNCC and NP/N samples) and the *NM*_*005654* isoform (46 kDa) in our gels (Fig. 7E and Supplementary Fig. 8D). Whether the weak additional bands of sizes other than 44–46 kDa present in hNCC but not in hiPSC (Ab1) may indicate degradation of NR2F1, or other unspecific proteins, remains to be elucidated, as this Ab has previously been shown to generate unspecific bands in mouse head tissue^103^. Bands present in both hNCC and hiPSC, especially when Ab2 and Ab7 were used (Fig. 7E) can be considered unspecific. While images of GAPDH used as a loading control were acquired uniformly with 20 s of exposure time for all seven membranes, different exposure times were required for each NR2F1-Ab (Supplementary Fig. 8A), although Ponceau S staining proved comparable protein transfer from the gel onto the nitrocellulose membrane (Supplementary Fig. 8B). Hence, similarly to IF (Figs. 3 and 4) or FC (Fig. 7A–D), this may also indicate that the WB conditions for each individual Ab could still be optimized.

The presence of unspecific bands in WB in our and previously published samples^62,103^ prompted us to systematically corroborate the problem using hNCC derived from *WT* hiPSC, hiPSC^−/−^ (Fig. 4), and from cells with heterozygous *NR2F1 KO* (hiPSC^+/−^). Again, different exposure times of the WB were required for the NR2F1-Abs, while they were the same for the loading control GAPDH (Supplementary Fig. 8A). Overall, five of the seven Abs (Abs1–5) detected a band corresponding to the size of the 44-46 kDa NR2F1 protein only in the *WT* and +/−, but not in −/− samples, implying their specificity (Fig. 7F, Supplementary Fig. 8E). In contrast to our previous results from hiPSC that lack NR2F1 expression (Fig. 7E), Ab6 and Ab7 detected a 44-46 kDa band also in hNCC^−/−^ samples. Considering that both Abs bind to the LBD of NR2F1 (Supplementary Fig. 4A), which shows very high homology between NR2F2 and NR2F1 (97%)^26,28,57,104^, we assume that these two Abs may recognize NR2F2 in addition to or instead of NR2F1, since both homologs are expressed in hNCC^32,69^ (Fig. 1, Supplementary Fig. 2B). This idea is further supported by the fact that after Ab-stripping of Ab2 and Ab4 and re-staining the same membranes with Ab6 and Ab7, a distinct 44–46 kDa band corresponding to the molecular weight of NR2F1/NR2F2 emerged in the hNCC^−/-^ (Supplementary Fig. 8C). However, for ultimate confirmation of possible NR2F1/NR2F2 Ab cross-reactivity in WB, further experimental evidence is required. Since all Abs detected one or more bands with molecular weights other than 44-46 kDa, albeit to varying degrees, we speculated that locally similar proteins identified by BLASTP (NR2F1 1-81 against which the Ab was raised) with described nucleolar localization^76^ may correspond to some of these unspecific bands. However, none of the reported molecular weights matched, except for Motor Neuron and Pancreas Homeobox Protein 1 (MNX1) with a molecular weight in the range of a lower unspecific band (40.6 kDa), which showed a local sequence similarity of 38.8% (Supplementary Fig. 3) and, importantly, is not expressed in hNCC^32,69^. Therefore, this protein cannot account for the nucleolar signal observed for Ab3 in nucleoli. Altogether, our findings underscore the need for proteomics approaches to elucidate this and other unspecific Ab signals.

## Discussion

Recently, it has been shown that many nucleolar proteins are also localized to other compartments, e.g., the nucleoplasm where they exert additional functions^75,76^. As a TF, NR2F1 is typically located in the nucleus^58–60^. However, its cytoplasmic or nucleolar localization has also been previously reported, although its potential physiological function in these compartments has not been explored^45,61–66^. Nonetheless, nucleolar-like NR2F1 staining detected in IF by Ab3 has been previously used to quantify NR2F1 in different tumor cells^61–66^. Architecture and assembly of nucleoli formed around specific chromosomal regions are highly dynamic. Nucleoli formation is regulated by phase separation, making them appear as liquid droplets within the nucleus^105^—a passive process involving weak intra- and intermolecular interactions between DNA, RNA, proteins, and lipids within a dynamic scaffold that, when disrupted, can lead to rare genetic diseases^67,106,107^ and regulation of ribosome number seems to be particularly important for early NP^108^. Given the nucleolar-like localization of NR2F1, we thought it might be directly involved in ribosome biogenesis. Thus, determining a potential non-canonical role of NR2F1 in nucleolar biology was of great interest, as it might have opened different perspectives for the study and possible treatment not only of cancer, but also of BBSOAS, Hirschsprung disease, Waardenburg syndrome type IV, and the development of peripheral blood and cardiac cells. Prompted by the nucleolar-like NR2F1 localization also observed in hiPSC-derived hNCC using Ab3, we systematically investigated this phenomenon by employing different human cell types, *NR2F1* overexpression, CRISPR/Cas9 engineered *NR2F1 knockout* cells and mouse embryonic brain tissue.

Consistent with previously reported results^45,61–63^, we initially showed that in hiPSC-derived hNCC endogenously expressing NR2F1, the nucleolar-like foci observed by Ab3 staining clearly colocalize with the nucleolar protein ZSCAN1^73^. However, we did not find NR2F1 in any of the available nucleolar protein databases^76,81,109,110^. Because these data lack hNCC information, we assumed that the nucleolar-like NR2F1 localization might be cell type-specific, as a cell type-dependent subcellular localization of the TF ZNF554 (Zinc Finger Protein 554) has been observed previously. In RT4 and SH-SY5Y cells, ZNF554 was found exclusively in the nucleoplasm, whereas in U2OS cells it was occasionally found in nucleoli^75^. However, we detected nucleoli-like NR2F1 foci not only in hNCC and HeLa cells endogenously expressing NR2F1 but also in hiPSC and HEK293 cells that do not express NR2F1. Moreover, we also detected these nucleoli-like NR2F1 foci in CRISPR/Cas9 engineered hiPSC^*−/−*^, raising further doubts about the nucleolar-like localization of NR2F1. Remarkably, our hiPSC-derived NP/N stained with Ab3 showed only sporadically nucleoli-like pattern of NR2F1. Moreover, in *WT* and *Nr2f1-null* mouse tissue, we did not observe such foci. Instead, we detected another unspecific staining, presumably of the extracellular matrix. Thus, our data strongly suggest that unspecific staining such as the nucleoli-like foci depends on the cell type and/or the procedure required for IF and makes using Ab3 for IF very questionable, in some experimental conditions.

To further support our, we analyzed the predicted protein properties of NR2F1. Like most nuclear receptors, the N-terminal domain of NR2F1 harboring amino acids 1-81 is annotated as disordered^84^ (Uniprot ID P10589). Proteins involved in nucleoli formation through liquid-liquid phase separation typically harbor intrinsically disordered domains, which have partly overlapping features with LCR^111^ and both features have been associated with nucleoli formation, among many other biological processes^105^. However, we did not observe amino acid enrichment typical for LCR^87,88^ or pronounced LCR in the self-similarity dot plot analysis. Moreover, employing a prediction tool for DNA-/RNA-binding probabilities of amino acids in protein sequences, no RNA-binding residues were predicted for either the shorter variant and the full-length variant, although a substantial fraction of nucleolar proteins is RNA-binding^112^. In addition, we found virtually no known nucleolarly localized proteins that have high protein sequence similarity with the NR2F1 isoforms using BLASTP, further reinforcing that NR2F1 is unlikely to be a nucleolar protein itself, and only very few of the known nucleolar proteins are potential interactors of NR2F1. Since co-expression of NR2F1 with these nucleolar NR2F1-interacting proteins is however not enough to explain the nucleolar signal in almost every hNCC, to date, we do not have any evidence about indirect binding of NR2F1 to nucleoli.

We noticed that the size and number of nucleolar-like foci stained with Ab3 appear stronger in HeLa cells and hNCC but weaker in NP/N, although all these cells endogenously express *NR2F1*. This is not surprising, since the composition, size, morphology and number of nucleoli are dynamic and differ between embryonic and differentiated cells^96,97^. We therefore speculate that in IF Ab3 recognizes a protein that is actually present in the nucleoli of some cell types only, but this would require further testing. For applications such as ChIP-seq, detection of nucleolar-like structures by Ab3 is less problematic as the profiles typically lack data on TF binding to ribosomal RNA gene chromatin due to their repetitive nature and exclusion from standard genome assemblies (Genome Reference Consortium^113^.

One useful way to verify actual protein localization is its tagging. A classic example of experimental evidence for the nucleolar localization of an otherwise nuclear protein LEO1 (RNA polymerase-associated protein), was previously obtained using a GFP-labeled cell line^76,114,115^. In addition, GFP-tagged chromatin architecture protein HMGB1 (High Mobility Group Protein B 1, a nonhistone nucleoprotein and an extracellular inflammatory cytokine) was shown to localize to the nucleoplasm in live imaging but to nucleoli after FA fixation^116^. However, hiPSC^*NR2F1-GFP*^ consistently showed exclusively nucleoplasmic but not nucleolar localization, as demonstrated by live imaging and staining with two different AbGFPs after FA fixation. In contrast, the GFP signal was distributed throughout all cell compartments in cells overexpressing GFP (hiPSC^*GFP*^). Overall, our data provide strong evidence that NR2F1 does not play a role in nucleoli, and strongly suggest that the nucleolar-like foci observed in IF may result from the binding of Ab3 to an unknown nucleolar target, rather than to the NR2F1 protein itself. This implies that, in certain cell types, there may be a nucleolar protein that carries a very similar sequence to the part of the NR2F1 protein against which the Ab was generated. However, as mentioned above, we did not identify a nucleolar protein that has high protein sequence similarity. To exclude the possibility of Ab3 detecting the nucleolar protein with the highest local sequence similarity (ZFHX4, 61.5% local sequence similarity), we found that this TF is typically not expressed in pluripotent stem cells such as hESC, making it unlikely to account for the Ab3 staining of nucleoli in virtually all hiPSC^32^. An example of confounding data resulting from IF visualization by an Ab was reported for nuclear speckles, membrane-less interchromatin space within the nucleus harboring RNA processing machinery and TFs but no DNA^117^. The Ab SC35 used in this study should principally recognize the splicing factor SRF2, but the main targets of this Ab are other proteins SRRM2 and SON, and band SRSF2 can be recognized to a lesser extent. Independent of revealing data misinterpretation using Ab SC35, the authors discovered additional roles of SRRM2 and SON in nuclear speckles formation. Hence, experimentally determining the potential non-NR2F1 target of Ab3 in nucleoli may still be worthwhile.

Since our results demonstrate that Ab3 is able to detect NR2F1 when it is highly expressed, e.g., in hiPSC^*NR2F1*^ and HEK293^*NR2F1*^ cells, we first assumed that the appearance of the nucleolar-like foci in *WT* hiPSC-derived hNCC could be explained by single-cell variability, as observed in U2OS cells for ZNF554^75^. Our previously published scRNA-seq data from hNCC^69^ confirmed that *NR2F1* expression is indeed quite heterogeneous and lower compared to e.g., *TFAP2A* and *NR2F2*. However, we also observed very distinct nucleolar-like staining in cells that do not express endogenous *NR2F1*, such as untransfected HEK293 and *WT* hiPSC and, even more strikingly, in hiPSC^*−/−*^. Therefore, we can exclude single-cell variability as a reason for the nucleolar-like foci observed by Ab3 and our data provide further evidence that the nucleolar-like foci are rather unspecific and only occur when *NR2F1* expression is below a certain level that remains to be determined.

One of the reasons for artifacts that may occur in IF may be related to cell fixation^3,4,116^. Therefore, we tested Ab3 in HeLa cells that endogenously express NR2F1, using different fixation types and times, and found that the nucleolar foci could be partially reduced by dehydration fixation and pre-clearing. To optimize dehydration conditions, we tested Ab3 in HEK293^*NR2F1*^ cells and found that no nucleolar-like foci appeared after fixation with 70% ethanol. As an organic solvent, ethanol can cause loss of small and soluble proteins, leading to speculation that the putative protein recognized by Ab3 e.g., in hiPSC^*−/−*^ that does not express NR2F1 may be small and/or soluble. It has been shown that PFA fixation efficiency depends on the amino acid sequence of the target protein^111,118,119^ and tertiary structures^120^ and that artifacts resulting from PFA fixation can lead to changes in the number, appearance, or disappearance of liquid condensates and thus to misinterpretation of experimental results^121^. It has been demonstrated that fixation can lead to artificial droplet-like foci in cells that otherwise have a homogeneous protein distribution within the nucleus with no visible spots in the live state^121^. We observed a partial improvement by the combination of ethanol with GA only in HeLa cells that were pre-cleared with Triton prior to the staining with Ab3. This could suggest that a soluble protein that is located in the nucleolus, and otherwise artificially stained by Ab3, might have been rinsed away. Pre-clearing with Triton is likely to preserve nuclear morphology, cell membrane integrity, and cytoplasmic structures, as shown by scanning electron microscopy of rat alveolar macrophages in vitro^122^. Both alcohol^123^ and FA^124^ are recommended as good fixation reagents, but our IF results clearly show that the choice is highly dependent on the Ab and target protein.

Encouraged by the effect of fixation with ethanol and elimination of the unspecific nucleolar-like foci in IF after using Ab3, we tested all Abs under these conditions in FC. This method allows high-throughput quantification and effective exclusion of background staining, which is not possible with IF, at least not to this extent. Using a pool of HEK293^*NR2F1*^ cells and a uniform fixation and staining protocol for all FC experiments, we demonstrated that, similar to IF, all Abs are in principle capable of recognizing NR2F1, although to a different extent. Taking advantage of the quantification and single-cell staining possible by FC, we showed that, under our conditions, Abs1, 3, and 4 recognize overexpressed NR2F1, ranging from low to high levels, whereas Abs2 and 5 showed only low signal, and Abs6 and 7 very weak NR2F1 signals. Direct visualization of the remaining stained cells under the fluorescence microscope further supports our FC results. Similar to IF, individual optimization of FC staining for Abs2, 5, 6, and 7, which was not possible in this study, could potentially improve NR2F1 staining.

Overall, our systematic comparison of all seven NR2F1 Abs provided by different vendors and targeting different regions of NR2F1 revealed that Ab1-4 are able to detect endogenous NR2F1/Nr2f1 in IF, but to different extents. Since these Abs target the N-terminus of NR2F1/Nr2f1, this region may be best suited for the production of specific NR2F1/Nr2f1 Abs. This region is also one of the least conserved among nuclear receptors and least homologous to NR2F2^26,84^. However, we only compared standard fixation and timing conditions for Ab3, so individual optimization for other Ab could improve IF staining of endogenous NR2F1/Nr2f1. Of note, under the same IF conditions, all Abs were able to detect highly expressed NR2F1 in HEK293^*NR2F1*^ cells, albeit also to different extents. These results lead us to speculate that manufacturers may have validated these Abs in an overexpression system rather than in a more physiologically relevant system. However, according to the datasheets (Supplementary Fig. 4B) most Abs were tested in cells or tissues endogenously expressing NR2F1. Hence, our data suggest that manufacturers should ideally consider multimodal validation approaches that do not preclude scientists from performing the necessary control experiments in their specific biological context before answering biological questions with these Abs.

In our last Ab-based assay, we systematically tested all NR2F1 Abs in WB under standard denaturing conditions that can alter the epitope^125,126^. To this end, we employed undifferentiated *WT* hiPSC and hNCC as well as hNCC^*+/-*^. When comparing *WT* hiPSC and hNCC, all seven Abs specifically recognized endogenous NR2F1 in hNCC and not in hiPSC, which do not express NR2F1 (nor NR2F2). However, besides the 44-46 kDa band corresponding to NR2F1, other bands with different sizes were also detected by all Abs, most of which were also present in hiPSC. Two of the seven Abs recognized a 44-46 kDa band also in hNCC^*−/−*^, likely corresponding to NR2F2 with high sequence homology, particularly in the LBD^60^. Since their expression patterns, targets, and functions partially overlap^32,127^, the high homology poses a major challenge for Ab development and specificity. Moreover, the predicted molecular size of NR2F2 is 43.5 kDa, and expression of both TFs has been reported in hNCC but not in hiPSC^69^ or hESC^32^. Therefore, we assume that the band visible in hNCC^−/−^ corresponds to NR2F2 and not NR2F1, although further experimental evidence is necessary to conclusively prove this. The hNCC differentiation of hiPSC carrying a double homozygous *KO* of *NR2F1* and *NR2F2*, if viable, would provide important information on the origin of this band. These questions highlight the challenges with Ab validation in general^128,129^.

Another question raised by our results is to what extent the isoforms *NM_001410754* and *ENST00000615873.1* can influence the performance of the N-terminal Abs. However, we have shown that both isoforms were recognized by all Abs tested here, although only *ENST00000615873.1* is endogenously expressed in our cells. Since the expression of most NR2F1 isoforms has not yet been functionally investigated, the development of isoform-specific Abs would certainly be of great benefit for this purpose.

As both monoclonal and polyclonal Abs have their advantages and disadvantages, we compared monoclonal mouse (Ab3) and rabbit (Abs1 and 4) as well as rabbit polyclonal Abs (Abs2, 5, 6 and 7). While monoclonal Abs can recognize and bind a specific epitope, polyclonal Abs can recognize and bind many different epitopes of a single antigen. Thus, monoclonal Abs are usually less likely to cross-react with other proteins than polyclonal Abs^130,131^. However, in the case of Ab3, we observed unspecific nucleolar foci in IF, and additional bands in WB that do not correspond to the molecular size of NR2F1 in hiPSC and hNCC^*−/−*^ that do not express *NR2F1*. Of note, Ab3 was the only mouse Ab we tested and the only one that produced unspecific nucleolar-like foci in IF. All other Abs were produced in rabbits, but some of them showed unspecific bands in the WB. It therefore remains to be determined whether these issues are related to the species in which they were generated or to the immunogens used for their generation. The summary of our results (Supplementary Table 1) can serve as a recommendation to the scientific community for the use of NR2F1 Ab and the method by which we were able to detect NR2F1 in our assays. In particular, Ab1 and Ab4 performed best in our hands, independently of sample type, fixation process or technique used.

There is a large number of publications on different fixation and permeabilization protocols for cells and tissues showing how important, but also how challenging it is to find the right Abs and suitable conditions^3,4,121,123,132,133^, and our work strongly confirms these findings. Several platforms have been developed (e.g., ENCODE project or Antibodypedia) that allow providers to post validation data in a standardized manner, and many efforts are being made to develop it further^134^. However, these platforms do not include all available Abs, and negative results are often not published. As a consequence, many scientists perform the same tests to find out that a certain Ab does not work under their conditions, which costs unnecessary time and money. Moreover, the lack of appropriate technical controls can lead to an inappropriate use of suboptimal Ab-based protocols, potentially compromising data interpretation. Therefore, it would be very helpful if scientists could share negative results using Abs in a specific context independent of the publications for which they were used. We are aware that the establishment of such a platform would need to be organized, and monitored, and the data would need to be easily updated and accessible. Such a platform would certainly be a logistical challenge, but very useful for the scientific community.

## Methods

### Cell culture

All cells were cultured under standard conditions at 37 °C in a 5% CO_2_ atmosphere. HeLa cells were grown in DMEM high glucose (Thermo Fisher Scientific, 11965092), supplemented with 1% Penicillin-Streptomycin (Thermo Fisher Scientific, 15070063) and 10% Fetal Bovine Serum (Biochrom, 511150). Human embryonic kidney (HEK293) cells were cultured in DMEM (Thermo Fisher Scientific 41965-039) supplemented with 10% inactivated Fetal Bovine Serum (FBS; ThermoFisher 10270-106). hiPSC (DPEDi001-A) used for NR2F1-GFP overexpression and hNCC differentiation were cultured on GelTrex (Thermo Fisher Scientific, A14132-02) coated dishes in StemMACS iPS-Brew XF (Miltenyl Biotec, 130-104-368), with medium changed every other day and cells were regularly split with Versene (GIBCO by Life Technologies, 15040066). hiPSC used for neuronal differentiation were maintained in StemFlex medium (Thermo Fisher Scientific; A3349401), on Matrigel (Corning 354234) coated cell culture plates. For routine passaging Accutase (Sigma A6964-100ML) was used and medium was supplemented with 10 µM Rock inhibitor (Y-27632; Stem Cell Technologies; #72304) for 24 h after passaging.

### Animals

We have complied with all relevant ethical regulations for animal use and all mouse experiments were conducted in accordance with relevant national and international guidelines and regulations (European Union rules; 2010/63/UE) and have been approved by the local ethical committee in France (CIEPAL NCE/2024-976). Homozygous *Nr2f1 KO* embryos were generated and genotyped as previously described^55^. Littermates of *KO* embryos with *WT Nr2f1* alleles were used as control mice. Midday of the day of the vaginal plug was considered as embryonic day 0.5 (E0.5). Control and mutant mice were bred in a 129S2/SvPas background. Both male and female embryos were used in this study; age is specified for each embryo used in specific experiments. Standard housing conditions were approved by the local ethical committee; briefly, adult mice were kept on a 12 h light-dark cycle and housed three per cage with the recommended environmental enrichment (wooden cubes, cotton pad, igloo) with food and water ad libitum.

### Generation of HEK239^*NR2F1*^ and hiPSC^*NR2F1-GFP*^

The full-length human NR2F1 sequence (ENST00000615873.1 equivalent to F1DAL9), was found in all our human cells expressing *NR2F1* and cloned into the pEGFP-C1 (Addgene-Plasmid #46956) and validated by Sanger sequencing and delivered into hiPSC using the non-liposomal FuGene HD transfection reagent (Promega, E2311) following the manufacturer’s instructions. 24 h after transfection, cells were subjected to IF assay. To generate HEK293^*NR2F1*^, the pcDNA3.1-NR2F1-DYK (*NM_005654*; Genscript clone ID: OHu23866D) vector with the full-length human *NR2F1* sequence was transfected using JetPRIME Polyplus (POL114-07), following manufacturer’s instructions. Two days after transfections, cells were detached by Trypsin-EDTA treatment (Thermo Fisher Scientific; 25200072) and either processed for FC or seeded on Matrigel-coated round glass coverslips in 24-well plates for IF.

### Generation of hiPSC^−/−^ and hiPSC^+/−^

hiPSC^−/−^ (PGP1 c2) and hiPSC^+/−^ (PGP1 F3) were generated by Synthego using CRISPR/Cas9 technology and guide RNA sequence: CUACGGCCAAUUCACCUGCG. For PCR amplification and sequencing (Forward primer: TGGCAATGGTAGTTAGCAGCT; Reverse primer: TTGAGGCACTTCTTGAGGCG) were used and clones with an indel mutation in exon 2 of *NR2F1* (exon 2 of ENST00000615873.2 or exon 1 of NM_005654.6) cut location hg38, chr5:93,585,331, causing frameshift in the DBD region and a premature stop codon which results in a truncated protein were picked and validated by Synthego. The absence of chromosomal anomalies was verified by using a PCR-based kit (Stem Cell Technologies; hiPSC Genetic Analysis Kit, # 07550).

### hiPSC-derived hNCC

To differentiate confluent hiPSC into hNCC^69^, the colonies were detached by 2 mg ml^−1^ collagenase, washed with PBS and plated in Petri dishes in hNCC differentiation medium (Neurobasal and DMEM F12 media in 1:1 ratio, 0.5x B27 with Vitamin A and 0.5x N2 supplements, 20 ng ml^−1^ epidermal fibroblast growth factor, 20 ng ml^−1^ basic fibroblast growth factor and 5 µg ml^−1^ Insulin) that was changed every 2–3 days. The resulting embryoid bodies (EB) typically attached at day 5–6 and gave rise to NCC outgrowths, which were harvested at day 11 using Accutase (Sigma-Aldrich, A6964). 50,000 cells per cm^2^ were seeded on cell culture dishes coated with 5 mg ml^−1^ fibronectin in hNCC maintenance medium in which insulin was replaced by 1 mg ml^−1^ BSA. Following one additional passage, hNCC cells were either seeded on round glass coverslips for IF or in six-well plates for WB and harvested in passage number 2.

### hiPSC-derived NP/N

We used a modified differentiation protocol based on dual inhibition of SMAD signaling^94^. Neural induction medium added to hiPSC at 80–90% confluency consisted of DMEM/F12 and supplemented with: N2 (Thermo Fisher Scientific; 17502-048), B27 (without vitamin A; Thermo Fisher Scientific; 12587-010), Glutamax (Thermo Fisher Scientific 35050-038), Na-Pyruvate (Sigma S8636), Penicillin–Streptomycin (Biozol ECL-ECB3001D) or Antibiotic/Antimycotic solution (Sigma A5955-100ML), β-Mercaptoethanol (0.05 mM; Thermo Fisher Scientific 31350-010), NEAA (1 mM; Thermo Fisher Scientific 11140-035) and Heparin (2 µg ml^−1^; Sigma H3149-25KU) and supplemented with LDN-193189 (0.25 µM; Sigma SML0559-5MG) and SB-431542 (5 µM; Sigma S4317-5MG). As soon as neural rosettes appeared (7–8 days after induction) the cells were cultured in the induction medium without LDN and SB for up to 1 month, by changing medium every other day. For redistributing cells prior to IF, cells were detached by Accutase (Sigma A6964-100ML) and seeded on Matrigel-coated round glass coverslips for one week.

### Life imaging and IF of human cells

We utilized the Leica DMIL LED fluorescence microscope and ×40 objective for live imaging of the GFP-transfected hiPSC. Cells used for IF were cultured on round glass coverslips in a medium appropriate for the cell type. hNCC, HeLa or undifferentiated *WT* hiPSC or transfected with *pEGFP-NR2F1* (hiPSC^*NR2F1-GFP*^) were fixed with 3.7% FA for 15 min, permeabilized with 0.5% Triton X-100 in PBS for 10 min and blocked with 5% BSA/PBS for 30 min at RT. The specific reagents and fixation times used for the optimization of the Ab3 staining in HeLa cells are indicated in the results. Primary Abs were incubated in 5% BSA/PBS overnight at 4 °C (see Supplementary Table 2 for additional information about the used Abs). Following three washing steps with PBS 0.05% Tween, secondary Abs diluted in 5% BSA/PBS were incubated for 30 min at 37 °C. Further details on the Abs used, including the concentrations used here, can be found in Supplementary Table 2. After three washing steps with PBS 0.05% Tween, the nuclei were stained with 1 µg ml^−1^ DAPI solution in PBS for 1 min at room temperature. Subsequently, the cells were rinsed three times with PBS and permanently mounted in Fluoromount-G (Life Technologies) and analyzed with a Leica DFC 7000T fluorescence microscope using ×40 objective with LAS X Software. Optionally, Keyence All-In-One fluorescent microscope BZ-X800, and the objectives x60 were used. The images were then assembled and labeled by ImageJ2 (using FiJi package) and edited by PhotoShop. HEK293 cells were fixed in 4% PFA (Sigma P6148-1KG) for 15 min at room temperature. The modified fixation times and reagents used for Ab3 staining optimization in HEK293 cells are indicated in the results. After fixation, cells were washed with PBS and blocked for 1 h with 5% sheep or fetal calf serum and 0.3% Tween or Triton diluted in PBS. Following incubation of primary Abs (4 h at RT or overnight at 4 °C), and three washing steps with 1% serum in PBS, secondary Abs were incubated at RT for 2-4 h. Hoechst or DAPI was included in the secondary Ab solution (10,000x dilution). Images were acquired with an Apotome Zeiss using ×10 and ×20 objectives by AxioVision software and edited by PhotoShop or by ImageJ/Fiji.

### IF of mouse embryonic brain sections

According to the Bio-Protocol^100^, mouse embryonic whole heads were dissected and fixed in 4% PFA (Sigma P6148-1KG) at 4 °C for 3–4 h in agitation, then washed in PBS and dehydrated in 25% sucrose overnight at 4 °C. After embedding in OCT resin at −80 °C, 12 µm-thick slices were obtained by cryostat sectioning and collected on SuperFrost slides (Thermo Fischer Scientific, Superfrost Plus: J1800AMNZ). Following antigen retrieval prior to incubation (10 min at 95 °C in pH = 6 Citric acid solution), the samples were blocked with 5% serum and 0.3% Tween or Triton in PBS for 1 h. The 1primary Ab were incubated for 4 h at RT or overnight at 4 °C and after subsequent three washing steps with PBS, the samples were incubated with adequate secondary Ab for 2–4 h at RT (Further details on the Abs used, including the concentrations used here, can be found in Supplementary Table 2). Hoechst or DAPI was included in the secondary Ab solution (10,000× dilution). For mounting, a solution of 80% glycerol and 2% N-propyl gallate was used. The images were acquired at an Apotome Zeiss using ×10 and ×20 objectives by AxioVision software and edited by PhotoShop.

### Flow cytometry

HEK293 cells were dissociated into a single cell suspension by using trypsin–EDTA and transferred into low-bind Eppendorf tubes, washed twice with ice-cold PBS and fixed with 70% ethanol while mixing on a vortexer, and stored at −20 °C. 200,000 cells were then stained in suspension using the same IF protocol as for cryostat sections, with the only difference being that a short centrifugation (5 min at 170×*g*) was used for the washing steps with 1% serum PBS. For each staining, 10,000 total events (excluding debris) from two independent batches (20,000 cells in total) were measured at the same voltage using the FITC (488) laser with the BD LSRFortessa system and FACSDiva software, and the data were then analyzed using FlowJo software (Becton Dickinson). Forward Scatter (FSC) and Side Scatter (SSC) plots were used to visualize and quantify the cell population selected for further examination (Cells), excluding cellular debris. To distinguish individual cells from aggregates or doublets, the Cells were additionally gated using Forward Scatter Height (FSC-H) versus Forward Scatter Area (FSC-A). The intensity of AF488 fluorescence was assessed using the FITC-A channel (NR2F1^total^). When the fluorescence intensity of positive cells exhibited a separation of more than one logarithmic unit from the negative population, Cells were categorized as having either low (NR2F1^low^) or high fluorescence levels (NR2F1^high)^. Data used in the analysis can be found in Supplementary Data 2.

### Western blot of hiPSC and hiPSC-derived hNCC

The cells were washed with PBS and incubated on ice for 1 h with ice-cold RIPA buffer (50 mM Tris–HCl, pH 7.5 with 150 mM sodium chloride, 1.0% NP-40, 0.5% sodium deoxycholate and 0.1% sodium dodecyl sulfate) supplemented protease and phosphatase inhibitor cocktail (100×) (Thermo Fischer Scientific #1861281). Following centrifugation for 30 min at 4 °C and at 13,000 rpm, the supernatant was collected, and the protein concentration was determined using Pierce™ BCA Protein Assay Kit (Thermo Fischer Scientific, 23225). Subsequently, 20 g of each sample was boiled in a Laemmli sample buffer (Bio-Rad) containing 50 mM β-mercaptoethanol. Finally, the proteins were separated by SDS–PAGE (NuPAGE 4–12%, Bis–Tris gel (Thermo Fischer Scientific, NP0322BOX) using XCell4 SureLock Mini-Cell Electrophoresis System (Thermo Fischer Scientific, EI0001) and transferred onto a nitrocellulose membrane (GE Healthcare Life science, 10600002) using the Mini-PROTEAN Tetra cell blotting device (BioRad, 1660827EDU). The membranes were blocked for 1 h with 5% BSA/PBS or 5% non-fat milk/TBS at RT and incubated overnight at 4 °C with primary Ab diluted in 5% BSA/PBS or 5% non-fat milk/TBS depending on manufacturer’s recommendation (see Supplementary Table 2 for additional information about the used Abs). After three washing steps with 0.05% Tween in PBS, the membranes were incubated for 1 h at RT with HRP Conjugate secondary Ab diluted in 5% BSA/PBS. Following three washing steps with 0.05% Tween in PBS the proteins were visualized by chemiluminescence using Clarity™ Western ECL Substrate kit (BioRad, 170-5060) and an INTAS ECL ChemoStar imaging system.

### Bioinformatical analyses

DNA/RNA-binding probabilities for all residues in the NR2F1 protein sequence were calculated by the DRNApred algorithm through the web-server interface^90^. Predictions of intrinsically disordered domains and IDP binding capacity based on the protein sequence were performed with IUPred3 and ANCHOR2 through the web–server interface^85^, using the “IUPred3 long disorder” option and medium smoothing. Protein BLAST (BLASTP) with NR2F1 1–81 (NM_005654) or 1–56 (ENST00000615873.1) as query sequence was run on UniProt. Gapped local sequence alignments (gap opening cost: 10, cap extension cost: (1) were performed against human proteins with a BLOSUM80 matrix. The resulting proteins (all with *E*-value < 0.01) corresponded to 118 unique genes. Described NR2F1-interacting proteins were collected from BioGRID, IntAct, MINT, and STRING. The overlap of both BLASTP results and the list of unique proteins physically interacting with NR2F1 with previously described nucleolar proteins^76^ was visualized using the *UpSetR* package^135^(1.4.0) in *R* (4.2.2) (R Core Team, 2022). DRNApred results were visualized with the *geom_tile()* function from *ggplot2* (3.4.1). The scRNA-seq dataset from hiPSC-derived NCC used in this study is based on publicly available data^69^. For visualization of *NR2F1* expression and some of its putative nucleolar interacting proteins, briefly, count data was preprocessed using standard *SingleCellExperiment* (1.20.0) workflow^136^ and *Seurat* (4.3.0)-based visualization^137^.

### Dot plot generation

Self-comparison dot plot of NR2F1was generated as described previously^87^ (for *NM_005654* with Uniprot ID: P10589 and for ENST00000615873.1 the F1DAL9 (protein database)). Briefly, every position in the NR2F1 protein sequence was compared to every other position in the protein in a 2D matrix, and matches were assigned foci.

### Statistics and reproducibility

IF data were statistically analyzed and graphically represented using Microsoft Office Excel software and GraphPad Prism (version 7.00). Quantitative data are shown as the mean standard error (SEM). For mean fluorescence intensity (MFI) quantification after IF, measurements were performed on at least 100 cells or on at least 6 brain areas, unless otherwise stated. To calculate the MFI, we used the FIJI in-built function “Adjust, Threshold” to detect nuclei/cells, followed by the “Analyze particles” and “Measure” functions to automatically quantify the pixel intensity in the areas of interest. For the mouse brain, Photoshop was used instead, by taking advantage of the “Magnetic Lasso” tool to manually select brain areas of interest, followed by the “Measurement” function to quantify the average fluorescence intensity inside the selected areas. The ratio of MFI for the sample of interest, i.e. NR2F1 overexpression or endogenously expressing tissue, and respective negative control (e.g., untransfected or *KO*) were calculated to obtain signal-to-noise ratios (SNRs) by dividing the average signal in the positive sample by the average signal in the negative controls/samples. Data used in the analysis can be found in Supplementary Data 1. Data were analyzed using the two-way ANOVA and statistical significance was set as follows: *p < 0.05; **p < 0.01; ***p < 0.001.

### Reporting summary

Further information on research design is available in the Nature Portfolio Reporting Summary linked to this article.

## Supplementary information

Supplementary Information

Description of Additional Supplementary Materials

Supplementary Data 1

Supplementary Data 2

Reporting Summary

## Data Availability

All data supporting our findings are included in the manuscript and supplementary files. Data used for the IF analysis for Figs. 3–5, as well as data used for the FC analysis for Fig. 7A–D are in the supplementary Excel data files. FC and WB data are available to the corresponding authors upon reasonable request. Publicly available data used in our study are cited in the manuscript and supplementary data, including human ChIP-seq data deposited into the GEO repository under accession numbers GSE28876 and GSE24447^32^ and scRNA-seq dataset^69^ deposited into the GEO repository under accession number GEO: GSE108522. The key resources table can be found in the Supplementary figures and tables.
