# Supplementary material for: Unravelling the conundrum of nucleolar NR2F1 localization using antibody-based approaches in vitro and in vivo
Source: Commun Biol. 2025 Apr 10;8:594. doi: 10.1038/s42003-025-07985-1 (PMC11982218; doi:10.1038/s42003-025-07985-1)
Supplement: Supplementary file 1 — Supplementary Information [file 42003_2025_7985_MOESM1_ESM.pdf]

Supplementary Figures and Tables:

Unravelling the conundrum of nucleolar NR2F1 localization using antibody-based approaches *in vitro* and *in vivo*.

Michele Bertacchi[1]*, Susanne Theiß[2], Ayat Ahmed[2], Michael Eibl[2], Agnès Loubat[1], Gwendoline Maharaux[1], Wanchana Phromkrasae[1], Krittalak Chakrabandhu[1], Aylin Camgöz[3,4], Marco Antonaci[2], Christian Schaaf[2], Michèle Studer[1], Magdalena Laugsch[2*]

[1] Université Côte d'Azur, CNRS, Inserm, Institute of Biology Valrose (iBV), 06108 Nice, France

[2] Institute of Human Genetics, Heidelberg University, Heidelberg, Germany

[3] Hopp Children's Cancer Center (KITZ), Im Neuenheimer Feld 280, 69120, Heidelberg, Germany.

[4] Division of Pediatric Neurooncology, German Cancer Research Center (DKFZ) and German Cancer Consortium (DKTK), Heidelberg, Germany

* Corresponding authors: Michele.BERTACCHI@univ-cotedazur.fr; Magdalena.Laugsch@uni-heidelberg.de

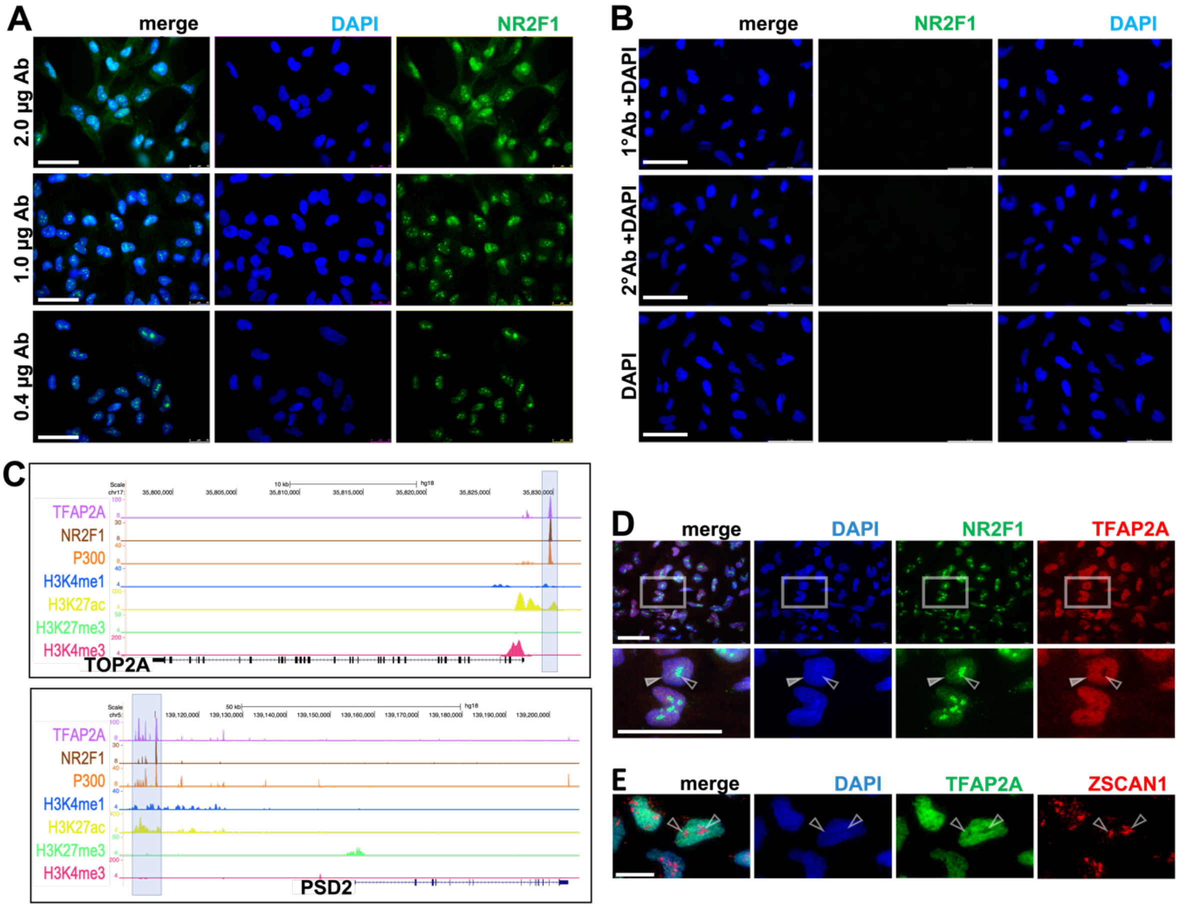

Supplementary Figure 1. Validation of endogenous NR2F1 IF-staining by Ab H8132 in hiPSC-derived hNCC, related to Figure 1: A) Testing of different Ab H8132 concentrations: 2 µg, 1 µg and 0.4 µg ml^{-1}. B) Control staining by sole primary Ab (Ab H8132) and sole secondary Ab anti-mouse AF488. C) UCSC Genome Browser view (hg18) of publically available ChIP-seq data[1] performed in hNCC derived from human embryonic stem cells (hESC) including Ab H8132 are originated from data deposited into GEO repository under accession numbers GSE28876 and GSE24447. Examples of genomic loci (*TOP2A* on chromosome 17 and *PSD2* on chromosome 5) with active enhancer chromatin states co-occupied by TFAP2A and NR2F1 (transparent blue bars). ChIP-seq signals for H3K27ac (yellow), H3K4me1 (green), and p300 (orange) map active enhancers that are co-occupied by TFAP2A (purple) and NR2F1 (brown). D) Co-IF of NR2F1 (green) and TFAP2A (red). Gray boxes indicate the selected zoom-in areas, empty grey arrowheads indicate aggregate-like clusters of NR2F1 without detectable TFAP2A signals. Full gray arrowheads indicate nucleoplasmic signals of both NR2F1 and TFAP2A. E) Co-IF of ZSCAN1 (red) with TFAP2A (green) showed no nucleolar-like co-localization. Empty arrowheads highlight aggregates observed only for NR2F1.Scale bars: 20 µm.

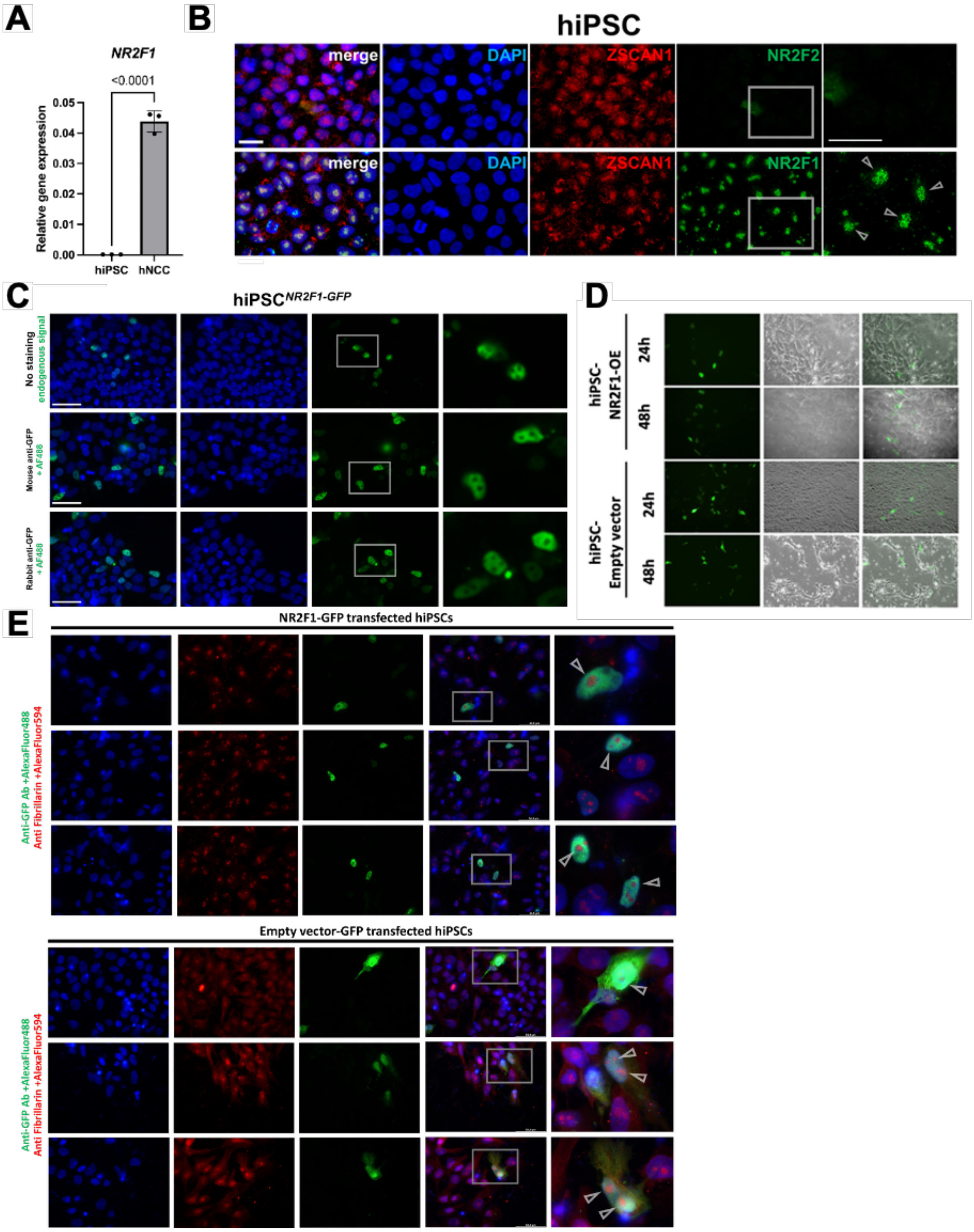

Supplementary Figure 2. Localization of endogenous and overexpressed NR2F1 in undifferentiated hiPSC, related to Figure 2: A) RT-qPCR data of the relative expression of *NR2F1* in *WT* hiPSC and hiPSC-derived hNCC showing that *NR2F1* is not detectable in hiPSC (unpaired t test, n=3). B) Co-IF of the nucleolar marker ZSCAN1 with NR2F1 or NR2F2 in hiPSC. The nucleolar marker ZSCAN1 (red) does not co-localize with NR2F2 (green), but with NR2F1 (green) when stained with by Ab H8132. C) hiPSC$^{NR2F1\text{-}GFP}$ (24 h post transfection) after fixation and staining with DAPI and after staining with anti-mouse or anti-rabbit AbGFP and DAPI. NR2F1-GFP is visible in the nucleoplasm and not in DAPI-depleted nucleolar foci. D) hiPSC$^{NR2F1\text{-}GFP}$, and hiPSC^{GFP} at 24h and 48h post transfection or hiPSC^{GFP} E) IF of hiPSC$^{NR2F1\text{-}GFP}$ (top three rows) and hiPSC^{GFP}, stained with AbGFP (green) and anti-Fibrillarin (red). Gary boxes indicate the selected zoom-in areas, empty grey arrowheads indicate nucleolar-like clusters of NR2F1 observed only by Ab H8132 staining. Scale bars: 20 µm in B, 50 µm.

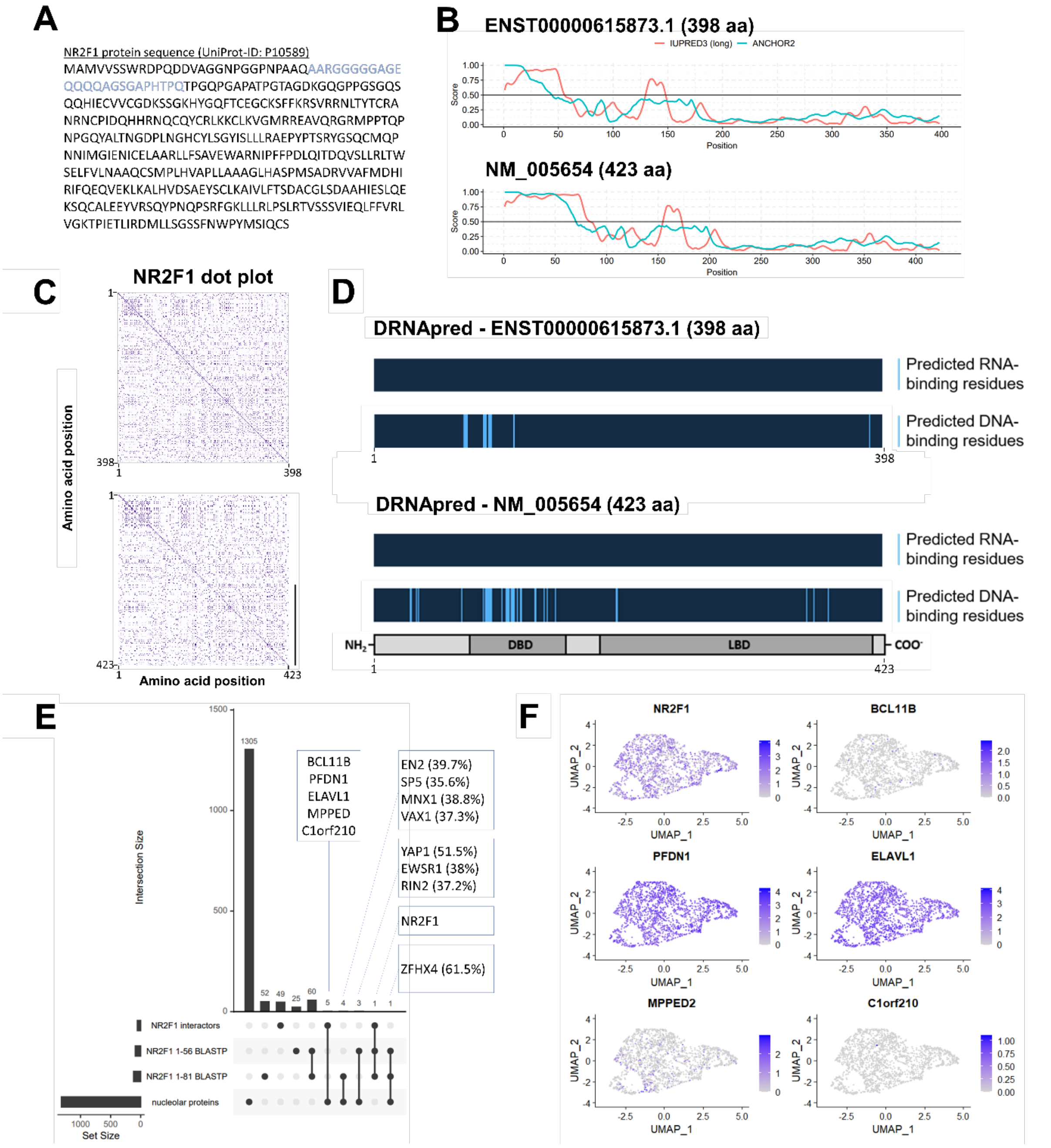

Supplementary Figure 3. Bioinformatical analysis of NR2F1 protein domains, putative interacting proteins and their expression in scRNA-seq, related to Figure 2: A) Amino acid (aa) sequence of the NR2F1 protein showing no enrichment of amino acids that are typically associated with LCRs, such as lysine and glutamic acid. The full sequence corresponds to the NM_005654 isoform (full-length NR2F1), the isoform ENST00000615873.1 corresponds to the same sequence without the amino acids marked in blue. B) IUPRED prediction of disordered regions in the protein sequence of NR2F1. The score as a measure for the level of disorder based on a biophysical model of intra-chain interactions along the full length of the protein sequence of the two isoforms shows some level of disorder only in the N-terminal region and the in the hinge domain between DBD and LBD. ANCHOR predicts the N-terminus as a possible disordered binding region. C) Self-comparison dot plot of both isoforms of NR2F1 protein sequences (ENST00000615873.1to the top, NM_005654 to the bottom). Every position in the NR2F1 protein sequences was compared to every other position in the proteins in a 2D-matrix. N-terminus to C-terminus is shown from top to bottom and left to right on the dot plot. Only very weakly dense regions appear along the diagonal in the N-termini of both variants, indicating some LCRs. D) DRNApred predicts enrichment of RNA-binding and DNA-binding residues in ENST00000615873.1 (top), NM_005654 (bottom). No RNA-binding residues were identified across the

entire proteins. Regardless of isoform, no RNA-binding residues were found. E) Upset plots indicate possibly nucleolar proteins with local sequence similarity to NR2F1 and their overlaps with possible NR2F1 interacting proteins. Human proteins showing sequences locally similar to the disordered domain of NR2F1 (either 1-56 or 1-81 for the short and long isoform, respectively) were identified with BLASTP. Their overlap with known nucleolar proteins is only marginal (proteins are indicated in the boxes) and had no higher local sequence similarity than 61.5%. Only five out of 1,308 known NR2F1-interacting proteins (grey circle) collected from BioGRID, IntAct, MINT, and STRING are localized to nucleoli. F) scRNA-seq data from Laugsch et al., 2019[2] confirmed that only a fraction of hiPSC-derived hNCC co-express NR2F1 and any of the five potential nucleolar interactors (<68%). Protein-protein interaction with any of these proteins is therefore unlikely to account for the nucleolar Ab H8132 signal, that we observed in virtually all hNCC (Figure 2A).

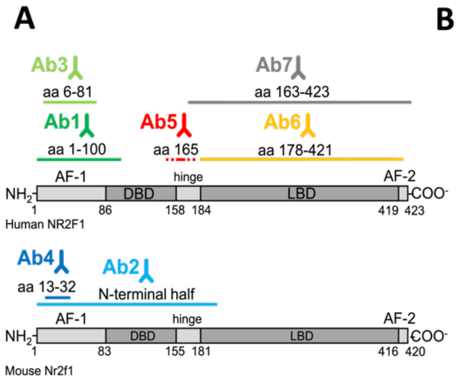

B

Number in this paper	Company	Ref number, clonality and host	Generation	Usage recommend-dation	Validation	RRID
Ab1	Abcam	ab181137 Rabbit-Monoclonal	Synthetic peptide	FC (intra), WB, IHC-P, ICC/IF	IHC: human breast carcinoma tissue; IF: Jurkat (Human T cell leukemia T lymphocyte) cells; WB: Human fetal brain lysates and Human fetal kidney lysates; FC (intra): MCF7 (Human cervix adenocarcinoma epithelial cells).	AB_2890250
Ab2	Sigma-Aldrich	abe1425 Rabbit-Polyclonal	GST-tagged recombinant protein	WB, IHC, IF, ChIP	IHC: rat cerebral cortex, human cerebellum, and human small intestine; IF: wild-type mouse E13.5 embryos and embryos with conditional brain region COUP-TF1-knockout; WB: Mouse brain tissue.	no
Ab3	Perseus Proteomics	PP-H8132-00 Mouse-Polyclonal	Recombinant human COUP-TFI	WB, IP, IHC, direct ELISA	-	AB_2155494
Ab4	Homemade-purified	Rabbit-Monoclonal	mouse Nr2f1 Sytheticpeptide sequence 13-32 (DDVAGGNPGGPNPAAQAARG)	-	-	no
Ab5	Cell Signaling Technology	6364 Rabbit-Monoclonal	Synthetic peptide	WB, and IP	Tested on various cell type for endogenous expression.	AB_11220432
Ab6	Proteintech	24573-1-AP Rabbit-Polyclonal	NR2F1 fusion protein Ag0118	WB, IHC, IF, ELISA	IHC: human ovary tissue; WB: human placenta tissue and NIH/3T3 (fibroblast cell line that was isolated from a mouse NIH/Swiss embryo).	AB_2879616
Ab7	Thermo Fisher Sicientic	PA5-30190 Rabbit-Polyclonal	Recombinant fragment corresponding to a region within aa 163 and 423 of Human COUP-TFI	WB, IP, ChIP	WB: transfected (–) and transfected (+) 293T whole cell (exogenous); ChIP: MCF7 (Human cervix adenocarcinoma epithelial cell) cells.	AB_2547664

Supplementary Figure 4. Overview of immunogen regions recognized by the anti-NR2F1 Abs tested in this study and complementary information, related to Figures 3-7. A) Grey: schematic structure and domains of the human NR2F1 (top) and the murine Nr2f1 protein (bottom). The immunogens used to produce the Abs are indicated in different colors above the NR2F1 domains used as antigen. B) Summary of available information, including clonality, host, and immunogen. IHC: immunohistochemistry; IHC-P: immunohistochemistry, paraffin; FC (intra): flow cytometry, intracellular; ELISA: enzyme-linked immunosorbent assay, RRID: Research Resource Identifiers. Note that all Abs are commercially available, with the exception of Ab4, which was a homemade one, produced and purified by Thermo Fischer Scientific using the mouse Nr2f1 peptide sequence 13-32 as an immunogen. Now available commercially, Ab1 has been produced as described elsewhere[3]. Importantly, the very high amino acid sequence homology between human NR2F1 and mouse Nr2f1 proteins, which share the same domains with only a few amino acids shifted, allows the use of the antibodies for both species[4].

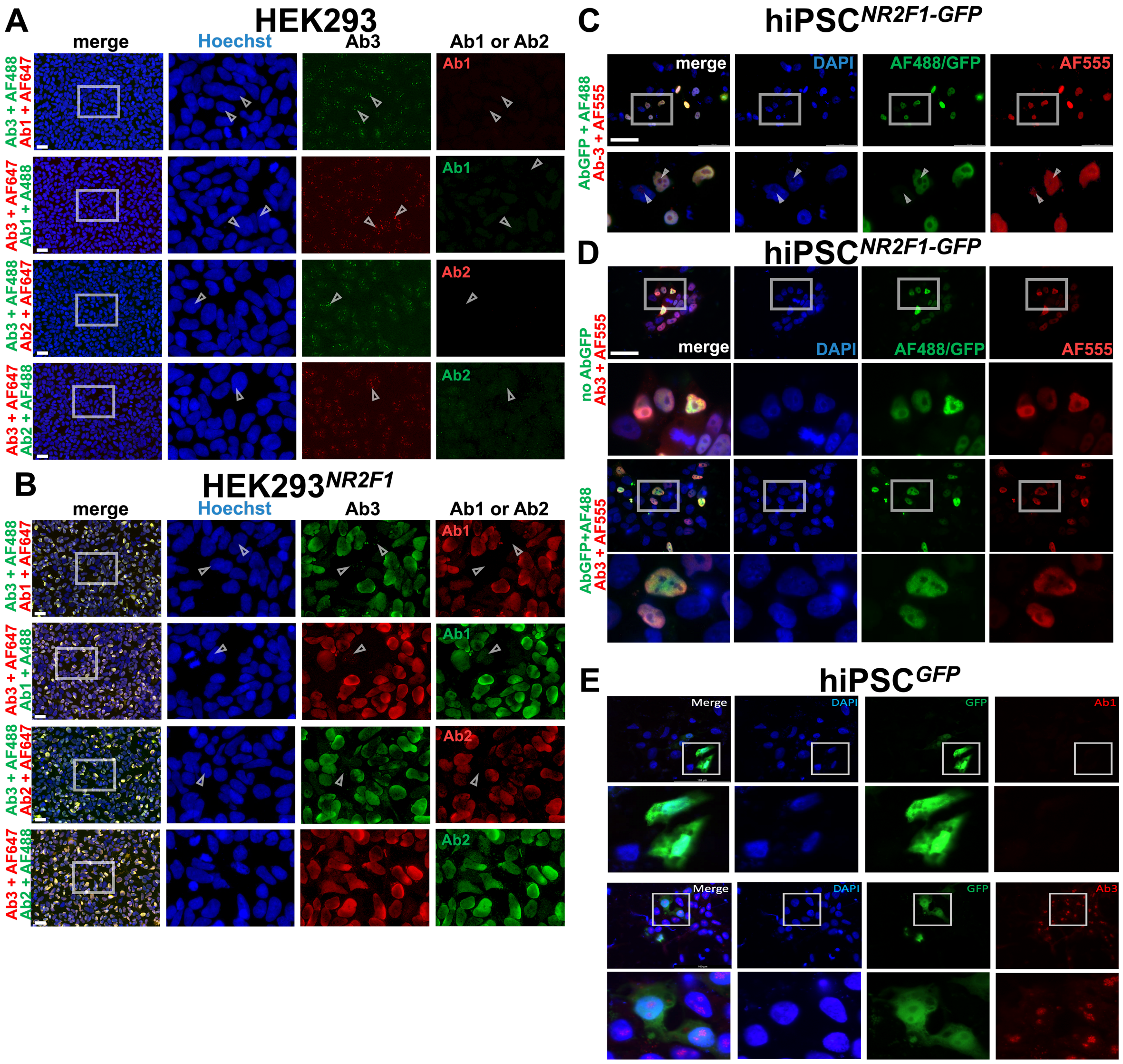

Supplementary Figure 5. Double IF staining of overexpressed NR2F1 in different cell types by Ab3 with Ab1 or Ab2, and different secondary Abs, related to Figure 3. Primary Abs and secondary Abs used are indicated on the left side of the pictures. Gary boxes indicate the selected zoom-in areas. A) Untransfected HEK293 cells co-stained with Ab3 and with either Ab1 or Ab2 in different combinations of adequate secondary Abs conjugated to AF647 (red) or AF488 (green). Arrowheads show nucleolar-like foci detected only in the Ab3 staining, regardless of which secondary Ab was used. B) In HEK293^{NR2F1} Ab1, Ab2 and Ab3 detected nuclear NR2F1 localization. But Ab3 stained in addition the nucleolar-like foci (arrowheads), apparently only in cells not successfully transfected with NR2F1, which were negative for Ab1 and Ab2 staining. C-D) hiPSC$^{NR2F1\text{-}GFP}$. C) Co-staining of Ab3 (red) with rabbit or mouse AbGFP and AF488 (green). Cells double-positive for GFP and Ab3 (yellow) display only a nuclear staining. GFP-negative (untransfected) cells show nucleolar-like foci stained by Ab3 (arrowheads). D) Cells stained with Ab1 only and co-stained with mouse AbGFP and rabbit Ab1 show overlapping and nucleoplasmic staining only. E) IF in hiPSC$^{NR2F1\text{-}GFP}$, stained with AbGFP (green) and Ab1 (red) do not show any signal, whereas Ab3 (red) results in signal in the nucleoli, which does not overlap GFP (green) signal. Nucleoplasm (blue) was stained with (A-B) Hoechst or (C-E) DAPI. Scale bars: 50 µm.

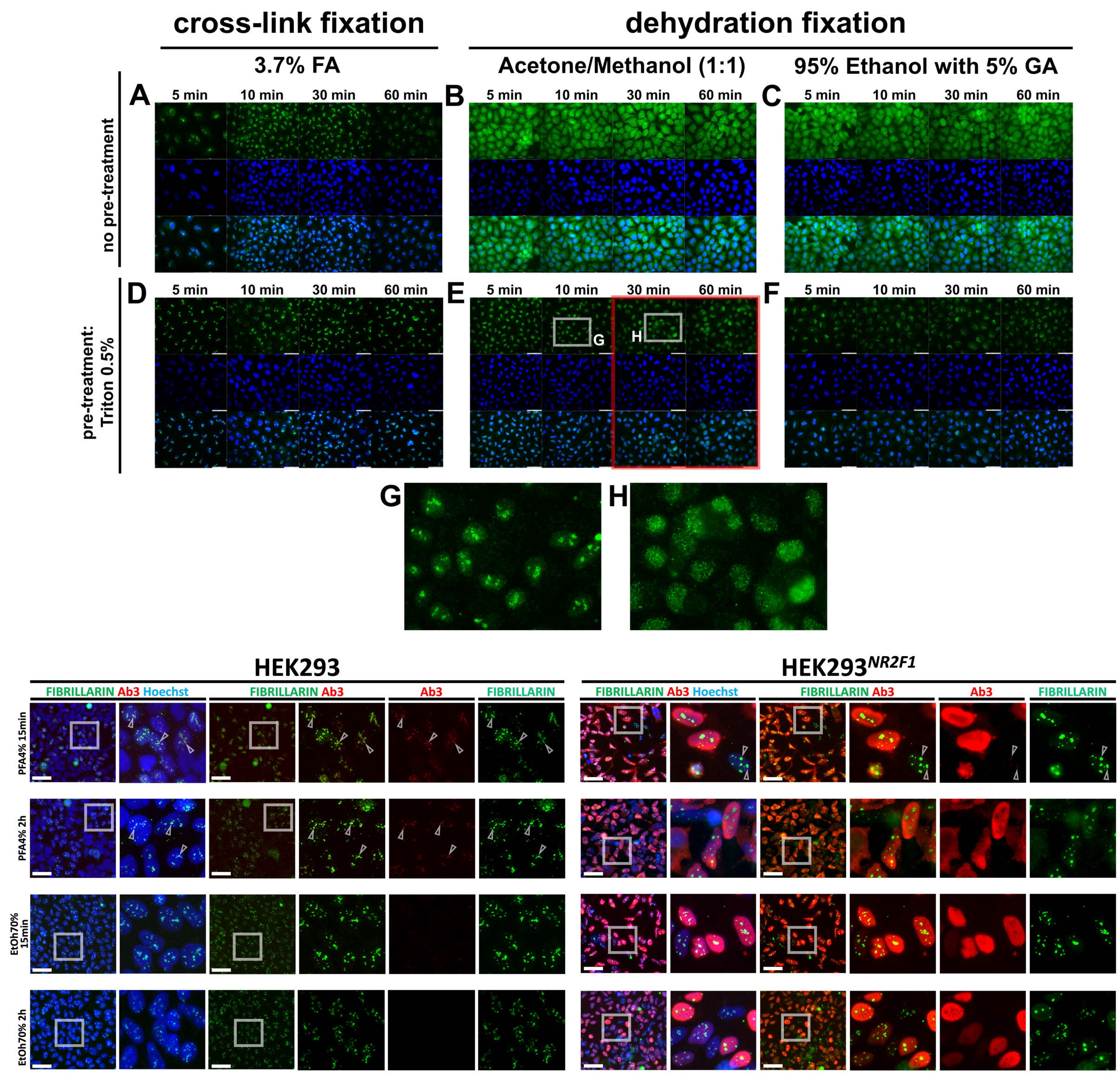

Supplementary Figure 6. Testing different fixation times and methods for endogenous NR2F1 in HeLa and HEK293 cells by IF using Ab3, related to Figure 6: Gray boxes show the selected zoom-in area placed next to it; arrowheads indicate nucleolar areas. (A-H) Pre-treatment with 0.5% Triton for 0, (A-C) or 30 seconds (D-F) is indicated at the left side. Fixation times of 5, 10, 30 and 60 minutes for FA are in A, D, G), for Acetone/methanol mix in 1:1 ratio in B, E) and for 95% ethanol supplemented with 5% GA in C, F). White squares in E show zoom-in of G) nucleolar-like pattern of NR2F1 at 5-10 minutes of acetone/methanol fixation and H) and 30 minutes resulting in nucleoplasmic NR2F1 localization consistent with staining typically resulting from IF with all other Abs. Nucleoplasm (blue) was stained with DAPI. Scale bars: 50 µm. I) Co-staining of Ab3 (red) with the nucleolar marker fibrillarin (green) in HEK293 and HEK293^{NR2F1} cells fixed with PFA fixation for 15 minutes confirm and complement data presented in Figure 6. Nuclei were stained by Hoechst. Scale bars: 50 µm.

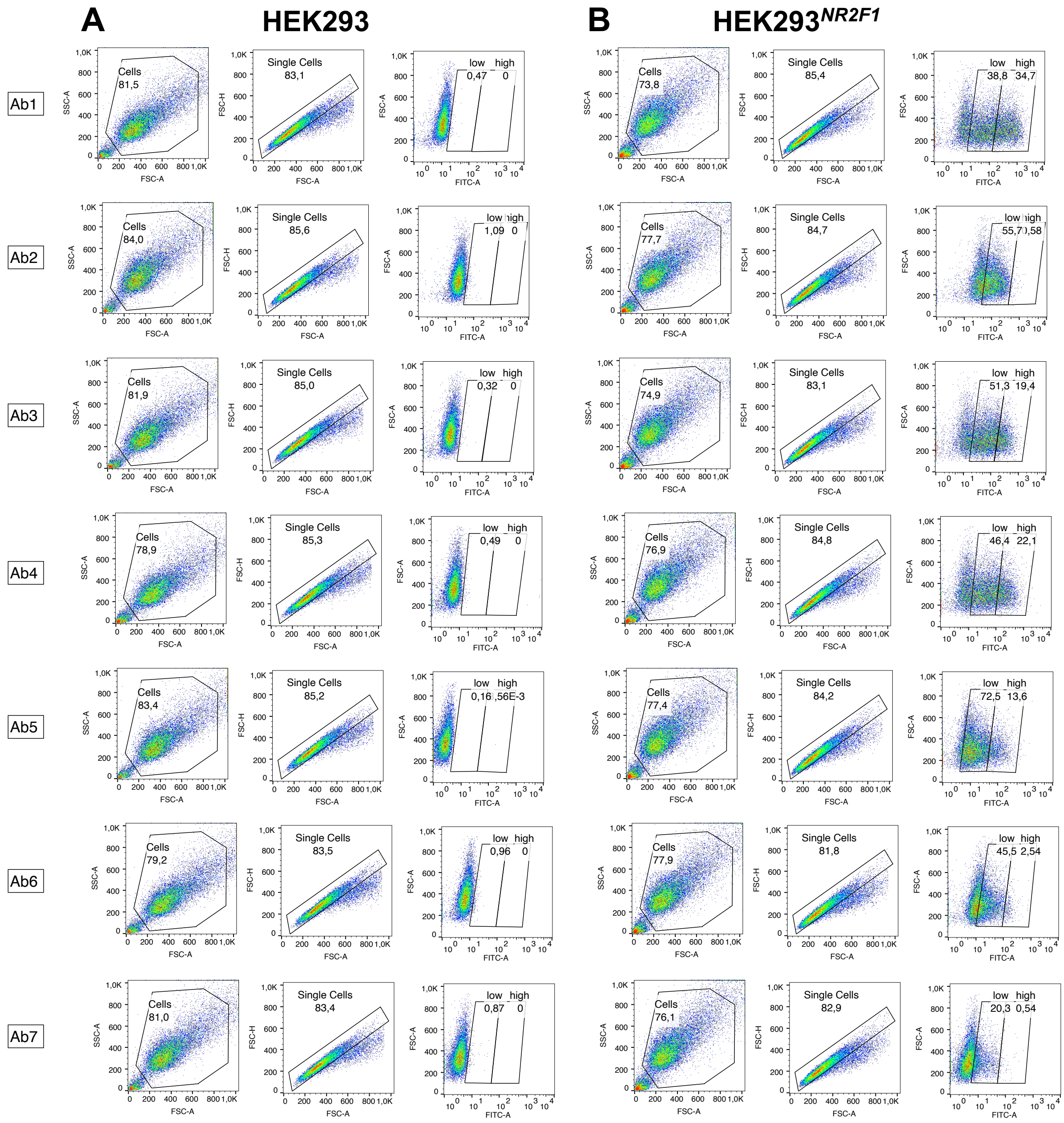

Supplementary Figure 7: Density plots and gating strategy applied for FC analysis related to related to Figure 7A-D. A) Untransfected HEK293 and B) HEK293*NR2F1* cells 48 h after transfection were fixed with 70% Ethanol, stained with the primary Abs1-7, and adequate secondary Ab AF488s. Forward Scatter (FSC) versus Side Scatter (SSC) plots show the population and percentage of cells (Cells) selected for downstream analysis excluding cell debris. Cells were plotted using Forward Scatter Height (FSC-H) versus Forward Scatter Area (FSC-A) to obtain Single Cells populations and exclude doublets. The AF488 fluorescence was measured by FITC-A by distinguishing between low ($HEK293^{low}$) and high ($HEK293^{high}$) levels of the fluorescence intensity based on when positive cells displayed more than one log separation.

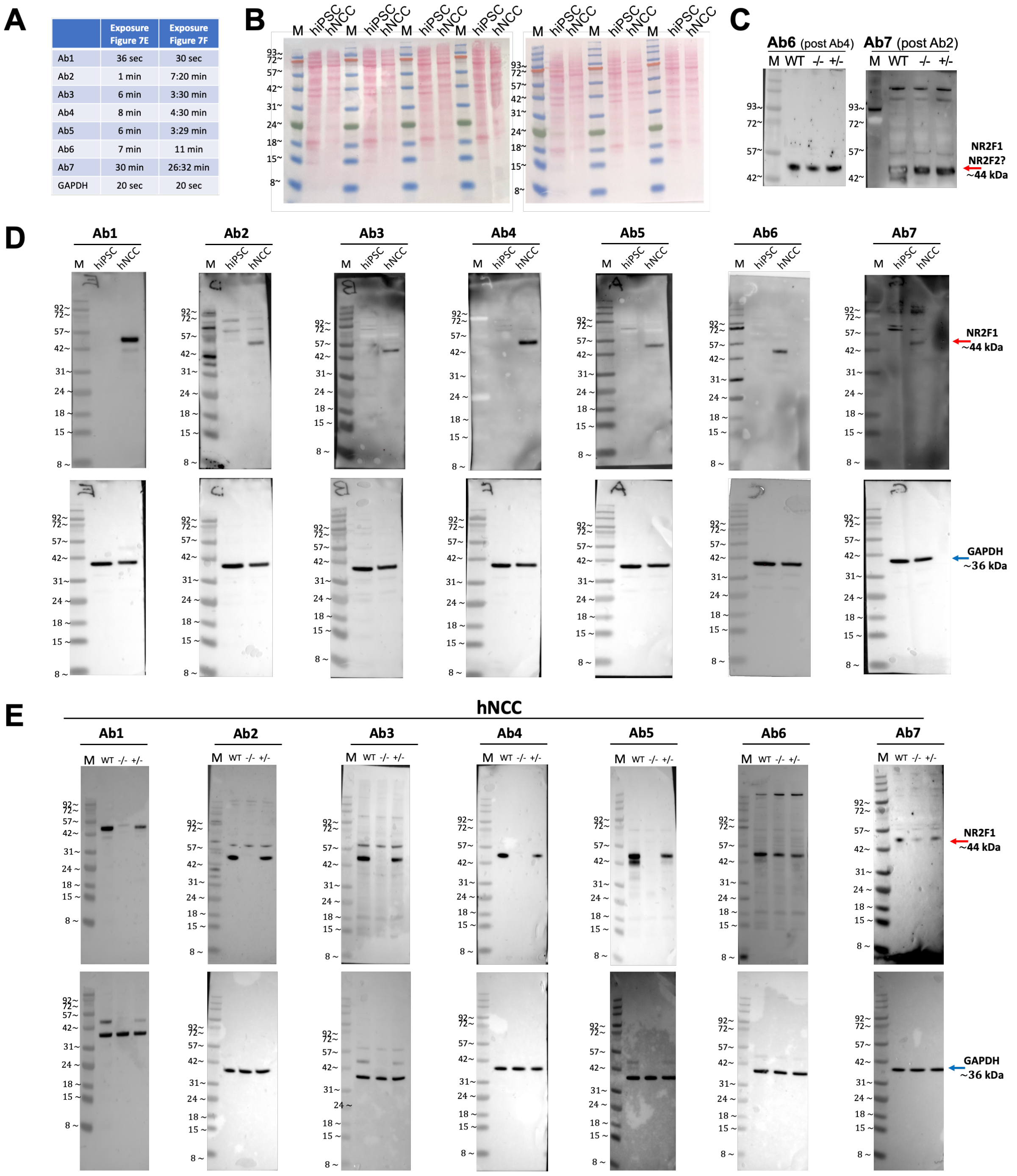

	Exposure Figure 7E	Exposure Figure 7F
Ab1	36 sec	30 sec
Ab2	1 min	7:20 min
Ab3	6 min	3:30 min
Ab4	8 min	4:30 min
Ab5	6 min	3:29 min
Ab6	7 min	11 min
Ab7	30 min	26:32 min
GAPDH	20 sec	20 sec

Supplementary Figure 8. Additional information about detection of NR2F1 in WB in *WT* hiPSC and hNCC as well as and hNCC$^{-/-}$ and hNCC$^{+/-}$, related to Figure 7E-F. 20 µg per sample of RIPA lysates were loaded. A) Exposure times used for each antibody in both WBs showed in Figure 7B) Ponceau S staining from WB showed in Figure 7E, which demonstrates an efficient protein transfer onto the membranes. C) Membranes stained with Ab2 and Ab4 in WB2 that did not show a 46 kDa band in hNCC$^{-/-}$ were stripped and stained again with Ab6 and Ab7, respectively. In hNCC$^{-/-}$, both Ab6 and Ab7 stained a 46 kDa band corresponding to the molecular weight of NR2F1/NR2F2. D) Uncropped membranes stained with all the seven NR2F1 Abs and the housekeeper GAPDH, showed in Figure 7E. E) Uncropped membranes stained with all the seven NR2F1 Abs and the housekeeper GAPDH, showed in Figure 7F.

Antibody	IF, standard, FA, hNCC	IF, standard, PFA, NP/N	IF, standard, PFA, NR2F1-transfected HEK293	IF, standard PFA 2 h mouse brain	FC, Ethanol fix NR2F1-transfected HEK293	WB hNCC
**Ab1** ab181137; rb/mono	***	***	***	***	***	***
**Ab2** abe1425; rb/poly	nd	**	***	**	*	*
**Ab3** H8132; m/mono	*	**	**	*	**	**
**Ab4** homemade; rb/mono	nd	**	**	**	***	***
**Ab5** #6364; rb/mono	**	-	**	-	*	*
**Ab6** 24573-1-AP; rb/poly	nd	-	*	-	*	NR2F2?
**Ab7** PA5-30190; rb/poly	nd	-	*	-	-	NR2F2?

Supplementary Table 1: Summary of the results of the individual Abs and assays used in this study under our conditions: *** = optimal, ** = good, * = sufficient, - = no signal; nd = not determined.

Abs used in this study	Company	Ref number	Conc. in T12 hiPSC, hNCC and HeLa cells	Conc.in HEK293 cells, PGP1 hiPSC, NP/N and mouse brain	Stock
Ab1	Abcam	ab181137-Rabbit-Monoclonal antibody	WB - 0.4 µg ml^{-1}l<br>IF - 1,6 µg ml^{-1}	0.4 µg ml^{-1}	0.4 µg μl^{-1}
Ab2	Sigma-Aldrich	abe1425-Rabbit-Polyclonal antibody	WB - 1:500	1:1000	not specified
Ab3	Perseus Proteomics	PP-H8132-00-Mouse-Polyclonal antibody	WB -1 µg ml^{-1}<br>IF - 4 µg ml^{-1}	1 µg ml^{-1}	1 µg μl^{-1}
Ab4	Homemade-purified	Rabbit-Monoclonal antibody	1:1000	1:1000	not specified
Ab5	Cell signaling	6364- Rabbit-Monoclonal antibody	WB - 1:1000<br>IF - 1:1000	1:1000	not specified
Ab6	Proteintech	24573-1-AP-Rabbit-Polyclonal antibody	WB - 0.6 µg ml^{-1}	0.4 µg ml^{-1}	0.4 µg μl^{-1}
Ab7	Thermo Fisher Scientific	PA5-30190-Rabbit-Polyclonal antibody	WB - 0.92 µg ml^{-1}	0,92 µg ml^{-1}	0.92 µg μl^{-1}
GAPDH	Abcam	ab8245- Mouse-Monoclonal antibody	WB - 0.4 µg ml^{-1}	not used	2 µg μl^{-1}
AbGFP Mouse	Santa Cruz	sc-9996- Mouse-Monoclonal antibody	IF - 20 µg ml^{-1}	not used	2 µg μl^{-1}
AbGFP Rabbit	Thermo Fisher Scientific	A-11122-Rabbit-Polyclonal antibody	IF - 1 µg ml^{-1}	not used	2 µg μl^{-1}
HRP Conjugate	BioRad	Goat Anti-Rabbit IgG (H + L)-#1706515	WB - 1:5000	not used	not specified
HRP Conjugate	BioRad	Goat Anti-Mouse IgG (H + L)-#1706516	WB - 1:5000	not used	not specified
AF488 Mouse	Thermo Fisher Scientific	# A-11001	IF - 2 µg ml^{-1}	2 µg ml^{-1}	2 µg μl^{-1}
AF488 Rabbit	Thermo Fisher Scientific	# A-11008	IF - 2 µg ml^{-1}	2 µg ml^{-1}	2 µg/ μl^{-1}
AF594 Mouse	Thermo Fisher Scientific	# A-11005	IF - 2 µg ml^{-1}	2 µg ml^{-1}	2 µg μl^{-1}
AF594 Rabbit	Thermo Fisher Scientific	# A-11012	IF - 2 µg ml^{-1}	2 µg ml^{-1}	2 µg μl^{-1}
TUJ1	Sigma-Aldrich	T8660	not used	1 µg/ ml^{-1}	1 µg μl^{-1}
DAPI	Thermo Fisher Scientific	# 62248	IF - 1 µg ml^{-1}	not used	1 µg μl^{-1}
Hoechst	Invitrogen	H3570	not used	1:10000	10 mg/ ml^{-1}

Supplementary Table 2: List of all antibodies and fluorescents staining DNA including concentrations used in all assays in this study.

Supplementary references:

1. Rada-Iglesias, A., Bajpai, R., Prescott, S., Brugmann, S.A., Swigut, T., and Wysocka, J. (2012). Epigenomic Annotation of Enhancers Predicts Transcriptional Regulators of Human Neural Crest. Cell Stem Cell *11*, 633–648. https://doi.org/10.1016/j.stem.2012.07.006.

2. Laugsch, M., Bartusel, M., Rehimi, R., Alirzayeva, H., Karaolidou, A., Crispatzu, G., Zentis, P., Nikolic, M., Bleckwehl, T., Kolovos, P., et al. (2019). Modeling the Pathological Long-Range Regulatory Effects of Human Structural Variation with Patient-Specific hiPSCs. Cell Stem Cell *24*, 736-752.e12. https://doi.org/10.1016/j.stem.2019.03.004.

3. Tripodi, M., Filosa, A., Armentano, M., and Studer, M. (2004). The COUP-TF nuclear receptors regulate cell migration in the mammalian basal forebrain. Development *131*, 6119–6129. https://doi.org/10.1242/dev.01530.

4. Alfano, C., Magrinelli, E., Harb, K., and Studer, M. (2014). The nuclear receptors COUP-TF: a long-lasting experience in forebrain assembly. Cell. Mol. Life Sci. *71*, 43–62. https://doi.org/10.1007/s00018-013-1320-6.

KEY RESOURCES TABLE

**REAGENT or RESOURCE**	**SOURCE**	**IDENTIFIER**
**Antibodies**		
Rabbit-Monoclonal anti COUP-TFI (NR2F1)	Abcam	Cat# ab181137; RRID: AB_2890250
Rabbit-Polyclonal anti COUP-TF I (NR2F1)	Sigma-Aldrich	Cat# abe1425
Mouse-Polyclonal anti COUP-TF1/NR2F1	Perseus Proteomics	Cat# PP-H8132-00; RRID: AB_2155494
Rabbit-Monoclonal anti COUP-TF I (NR2F1)	Homemade-purified	N/A
Rabbit-Monoclonal anti COUP-TF I (NR2F1)	Cell signaling	Cat# 6364; RRID: AB_11220432
Rabbit-Polyclonal anti NR2F1	Proteintech	Cat# 24573-1-AP; RRID: AB_2879616
Rabbit-Polyclonal anti NR2F1	Thermo Fisher Scientific	Cat# PA5-30190; RRID: AB 2547664
Mouse-Monoclonal anti GAPDH	Abcam	Cat# ab8245; RRID: AB_2107448
Mouse- Monoclonal anti GFP	Santa Cruz Biotechnology	Cat# sc-9996; RRID: AB_627695
Rabbit-Polyclonal anti GFP	Thermo Fisher Scientific	Cat# A-11122; RRID: AB_221569
Rabbit- Polyclonal anti Fibrillarin	Thermo Fisher Scientific	Cat# PA5-143604; RRID: AB_2942833
Mouse- Monoclonal anti Fibrillarin	Thermo Fisher Scientific	Cat# MA3-16771; RRID:AB_2105791
Mouse- Monoclonal anti NPM1	Thermo Fisher Scientific	Cat# 32-5200; RRID: AB_2533084
Goat- Polyclonal anti NPM1	Perseus Proteomics	Cat# AF5205; RRID: AB_2155176
Goat Anti-Rabbit IgG (H + L)	BioRad	Cat# 1706515
Goat Anti-Mouse IgG (H + L)	BioRad	Cat# 1706516
Goat anti-Mouse IgG (H+L) Cross-Adsorbed Secondary Antibody, Alexa Fluor™ 488	Thermo Fisher Scientific	Cat# # A-11001; RRID: AB_2534069
Goat anti-Rabbit IgG (H+L) Cross-Adsorbed Secondary Antibody, Alexa Fluor™ 488	Thermo Fisher Scientific	Cat# # A-11008; RRID: AB_143165

Goat anti-Mouse IgG (H+L) Cross-Adsorbed Secondary Antibody, Alexa Fluor™ 594	Thermo Fisher Scientific	Cat# # A-11005; RRID: AB_2534073
Goat anti-Rabbit IgG (H+L) Cross-Adsorbed Secondary Antibody, Alexa Fluor™ 594	Thermo Fisher Scientific	Cat# A-11012; RRID: AB_2534079
Mouse-Monoclonal Anti-β-Tubulin III	Sigma-Aldrich	Cat# T8660, RRID: AB_477590
DAPI Nucleic Acid Stain	Thermo Fisher Scientific	Cat# # 62248
Hoechst Nucleic Acid Stain	Thermo Fisher Scientific	Cat# H3570
**Bacterial and virus strains**		
E. coli Top10	Kurian Lab	N/A
**Chemicals, peptides, and recombinant proteins**		
GelTrex	Thermo Fisher Scientific	Cat#A1413302
Matrigel	Corning	Cat#354234
StemFlex medium	Thermo Fisher Scientific	Cat# A3349401
StemMACS iPS-Brew XF	Miltenyl Biotec	Cat#130-104-368
Versene	Gibco	Cat#15040-066
Accutase	Sigma-Aldrich	Cat#A6964
Trypsin-EDTA	Thermo Fisher Scientific	Cat# 25200072
Rock inhibitor (Y-27632)	Stem Cell Technologies	Cat# #72304
Thiazovivin	Axon Med Chem	Cat#1535
Human Plasma Fibronectin Purified Protein	Millipore	Cat#FC010
Neurobasal medium	Thermo Fisher Scientific	Cat#21103049
DMEM F12 medium	Thermo Fisher Scientific	Cat#10565018
B27 supplement	Thermo Fisher Scientific	Cat#17504044
N2 supplement	Thermo Fisher Scientific	Cat#17502048
DMEM high glucose	Thermo Fisher Scientific	Cat#11965092
Penicillin-Streptomycin	Thermo Fisher Scientific	Cat# 15070063
Fetal Bovine Serum	Biochrom	Cat# 511150
Animal-Free Recombinant Human EGF	Peprotech	Cat# AF-100-15
Recombinant Human FGF-basic (154 a.a.)	Peprotech	Cat# 100-18B
Insulin	Sigma-Aldrich	Cat# 11376497001
Glutamax	Thermo Fisher Scientific	Cat# 35050-038
Na-Pyruvate	Sigma-Aldrich	Cat# S8636
β-Mercaptoethanol	Thermo Fisher Scientific	Cat# 31350-010
NEAA	Thermo Fisher Scientific	Cat# 11140-035
Heparin	Sigma-Aldrich	Cat# H3149-25KU
LDN-193189	Sigma-Aldrich	Cat# SML0559-5MG

SB-431542	Sigma-Aldrich	Cat# S4317-5MG
Fluoromount-G™ Mounting Medium	Thermo Fisher Scientific	Cat# 00-4958-02
Paraformaldehyde	Sigma-Aldrich	Cat# 6148
Protease & phosphatase inhibitor cocktail (100x)	Thermo Fischer Scientific	Cat# 1861281
4x Laemmli sample buffer	Bio-Rad	Cat# 1610747
SDS-PAGE (NuPAGE 4-12%, Bis-Tris gel)	Thermo Fischer Scientific,	Cat# NP0322BOX
CozyHi™ Prestained Protein Ladder	HiQue	Cat# #PRL0202c1
Clarity™ Western ECL Substrate kit	BioRad	Cat# 170-5060
**Critical commercial assays**		
JetPRIME transfection reagent	Polyplus	Cat# POL114-07
FuGene HD transfection reagent	Promega	Cat#E2313
hiPSC Genetic Analysis Kit	Stem Cell Technologies	Cat# # 07550
Pierce™ BCA Protein Assay Kit	Thermo Fischer Scientific	Cat# 23225
innuPREP DNA/RNA Mini Kit	Analytik Jena	Cat# 845-KS-20800250
ProtoScript II First Strand cDNA Synthesis Kit	New England Biolabs	Cat# E6560L
PureYield™ Plasmid Miniprep System	Promega	Cat# A2495
**Experimental models: Cell lines**		
Human: WT hiPSC line T12- DPEDi001-A	Pediatric Endocrinology and Diabetology, Department of Pediatrics, University Clinic, Dresden, Germany	N/A
Human: WT hiPSC line PGP1	Synthego	N/A
Human: NR2F1-/- hiPSC line PGP1- C1	Synthego	N/A
Human: NR2F1+/- hiPSC line PGP1- F3	Synthego	N/A
HEK293 cell line	Université Côte d'Azur, CNRS, Inserm, Institute of Biology Valrose (iBV), 06108 Nice, France	N/A
Hela cells	Institute of Physiological Chemistry, Medical Faculty Carl Gustav Carus, Dresden University of Technology, Dresden, Germany	N/A
**Experimental models: Organisms/strains**		
COUP-TFI^{null} mouse line	Armentano et al., 2006	
**Oligonucleotides**		
gRNA-NR2F1 point mutation	CUACGGCCAAUUCA CCUGCG	
PCR_NR2F1_PointMutation_FW	TGGCAATGGTAGTTA GCAGCT	
PCR_NR2F1_PointMutation_RV	TTGAGGCACTTCTT GAGGCG	

qPCR_NR2F1_FW	TGCCTCAAAGCCAT CGTGCTGT	
qPCR_NR2F1_RV	CAGCAGCAGTTTGC CAAAACGG	
qPCR_ACTB_FW	TCAAGATCATTGCTC CTCCTGAG	
qPCR_ACTB_RV	ACATCGCTGGAAGG TGGACA	
qPCR_GAPDH_FW	GCACCGTCAAGGCT GAGAAC	
qPCR_GAPDH_RV	AGGGATCTCGCTCC TGGAA	
**Recombinant DNA**		
pEGFP-C1 Plasmid	Addgene	Addgene# 46956
pEGFP-C1-NR2F1 Plasmid	This paper	N/A
pcDNA3.1	Thermo Fisher Scientific	Cat# V79020
pcDNA3.1-NR2F1-DYK	This paper	
**Software and algorithms**		
R (4.2.2)	R Core Team, 2022	https://www.R-project.org/
ImageJ2	Schneider et al. 2012	https://imagej.nih.gov/ij/
Adobe PhotoShop	Adobe	
FlowJo software	Becton Dickinson	
DRNApred algorithm	Krugen lab[88]	http://biomine.cs.vcu.edu/servers/DRNApred/
Protein BLAST	BLASTP	https://www.uniprot.org/blast
