# Supplementary material for: Unravelling the conundrum of nucleolar NR2F1 localization using antibody-based approaches in vitro and in vivo
Source: Commun Biol. 2025 Apr 10;8:594. doi: 10.1038/s42003-025-07985-1 (PMC11982218; doi:10.1038/s42003-025-07985-1)
Supplement: Supplementary file 2 — Description of Additional Supplementary Materials [file 42003_2025_7985_MOESM2_ESM.pdf]

Description of Additional Supplementary Files

File name: Supplementary Data 1

Description: IF Raw Data used for statistical analysis graphically represented in Figure 3 C-D, Figure 4 D, E and F, as well as Figure 5 G-H.

File name: Supplementary Data 2

Description: Row Data used for FC analysis using FlowJo software showed in Figure 7 A-D and Supplementary Figure 7
