# Supplementary material for: Unravelling the conundrum of nucleolar NR2F1 localization using antibody-based approaches in vitro and in vivo
Source: Commun Biol. 2025 Apr 10;8:594. doi: 10.1038/s42003-025-07985-1 (PMC11982218; doi:10.1038/s42003-025-07985-1)
Supplement: Supplementary file 5 — Reporting Summary [file 42003_2025_7985_MOESM5_ESM.pdf]

nature portfolio

Corresponding author(s): Magdalena.Laugsch@uni-heidelberg.de
Michele.BERTACCHI@univ-cotedazur.fr

Last updated by author(s): 21.02.2025

Reporting Summary

Nature Portfolio wishes to improve the reproducibility of the work that we publish. This form provides structure for consistency and transparency in reporting. For further information on Nature Portfolio policies, see our Editorial Policies and the Editorial Policy Checklist.

Statistics

For all statistical analyses, confirm that the following items are present in the figure legend, table legend, main text, or Methods section.

n/a	Confirmed	
☐	☒	The exact sample size (*n*) for each experimental group/condition, given as a discrete number and unit of measurement
☐	☒	A statement on whether measurements were taken from distinct samples or whether the same sample was measured repeatedly
☐	☒	The statistical test(s) used AND whether they are one- or two-sided<br>*Only common tests should be described solely by name; describe more complex techniques in the Methods section.*
☒	☐	A description of all covariates tested
☒	☐	A description of any assumptions or corrections, such as tests of normality and adjustment for multiple comparisons
☒	☐	A full description of the statistical parameters including central tendency (e.g. means) or other basic estimates (e.g. regression coefficient) AND variation (e.g. standard deviation) or associated estimates of uncertainty (e.g. confidence intervals)
☒	☐	For null hypothesis testing, the test statistic (e.g. *F*, *t*, *r*) with confidence intervals, effect sizes, degrees of freedom and *P* value noted<br>*Give P values as exact values whenever suitable.*
☒	☐	For Bayesian analysis, information on the choice of priors and Markov chain Monte Carlo settings
☒	☐	For hierarchical and complex designs, identification of the appropriate level for tests and full reporting of outcomes
☒	☐	Estimates of effect sizes (e.g. Cohen's *d*, Pearson's *r*), indicating how they were calculated

Our web collection on statistics for biologists contains articles on many of the points above.

Software and code

Policy information about availability of computer code

Data collection	BioGR, IntAct, MINT, STRING,
Data analysis	http://biomine.cs.vcu.edu/servers/DRNApred/, https://iupred3.elte.hu/, https://www.uniprot.org/blast, https://www.R-project.org

For manuscripts utilizing custom algorithms or software that are central to the research but not yet described in published literature, software must be made available to editors and reviewers. We strongly encourage code deposition in a community repository (e.g. GitHub). See the Nature Portfolio guidelines for submitting code & software for further information.

Data

Policy information about availability of data

All manuscripts must include a data availability statement. This statement should provide the following information, where applicable:

- Accession codes, unique identifiers, or web links for publicly available datasets
- A description of any restrictions on data availability
- For clinical datasets or third party data, please ensure that the statement adheres to our policy

human ChIP-seq data fromGSE28876, GSE24447; scRNA-seq data from GSE108522

Research involving human participants, their data, or biological material

Policy information about studies with human participants or human data. See also policy information about sex, gender (identity/presentation), and sexual orientation and race, ethnicity and racism.

Reporting on sex and gender	N.A.
Reporting on race, ethnicity, or other socially relevant groupings	N.A.
Population characteristics	N.A.
Recruitment	N.A.
Ethics oversight	N.A.

Note that full information on the approval of the study protocol must also be provided in the manuscript.

Field-specific reporting

Please select the one below that is the best fit for your research. If you are not sure, read the appropriate sections before making your selection.

☒ Life sciences ☐ Behavioural & social sciences ☐ Ecological, evolutionary & environmental sciences

For a reference copy of the document with all sections, see nature.com/documents/nr-reporting-summary-flat.pdf

Life sciences study design

All studies must disclose on these points even when the disclosure is negative.

Sample size	All experiments were pferormed at least in triplicate, unless specified. For IF, at leasr 100 cells per sample were analysed. For FC, at least 10.000 events were measured
Data exclusions	No data were exluded
Replication	All attempts to replication were successful
Randomization	All samples were allocated in the group accroding to genetic background or experimental treatment
Blinding	Since all data were analysed the same way, blinding was not necessary

Behavioural & social sciences study design

All studies must disclose on these points even when the disclosure is negative.

Study description	N.A.
Research sample	N.A.
Sampling strategy	N.A.
Data collection	N.A.
Timing	N.A.
Data exclusions	N.A.
Non-participation	N.A.
Randomization	N.A.

Ecological, evolutionary & environmental sciences study design

All studies must disclose on these points even when the disclosure is negative.

Study description	N.A.
Research sample	N.A.
Sampling strategy	N.A.
Data collection	N.A.
Timing and spatial scale	N.A.
Data exclusions	N.A.
Reproducibility	N.A.
Randomization	N.A.
Blinding	N.A.

Did the study involve field work? ☐ Yes ☒ No

Field work, collection and transport

Field conditions	N.A.
Location	N.A.
Access & import/export	N.A.
Disturbance	N.A.

Reporting for specific materials, systems and methods

We require information from authors about some types of materials, experimental systems and methods used in many studies. Here, indicate whether each material, system or method listed is relevant to your study. If you are not sure if a list item applies to your research, read the appropriate section before selecting a response.

Materials & experimental systems

n/a	Involved in the study	
☐	☒	Antibodies
☐	☒	Eukaryotic cell lines
☒	☐	Palaeontology and archaeology
☐	☒	Animals and other organisms
☒	☐	Clinical data
☒	☐	Dual use research of concern
☒	☐	Plants

Methods

n/a	Involved in the study	
☒	☐	ChIP-seq
☐	☒	Flow cytometry
☒	☐	MRI-based neuroimaging

Antibodies

Antibodies used

Abcam ab181137-Rabbit-Monoclonal antibody
Sigma-Aldrich abe1425-Rabbit-Polyclonal antibody
Perseus Proteomics PP-H8132-00- Mouse-Polyclonal antibody
Homemade-purified Rabbit-Monoclonal antibody
Cell signaling 6364- Rabbit-Monoclonal antibody
Proteintech 24573-1-AP- Rabbit-Polyclonal antibody
Thermo Fisher Scientific PA5-30190- Rabbit-Polyclonal antibody
Abcam ab8245- Mouse-Monoclonal antibody

Santa Cruz sc-9996- Mouse- Monoclonal antibody
Thermo Fisher Scientific A-11122-Rabbit-Polyclonal antibody
BioRad Goat Anti-Rabbit IgG (H + L)-#1706515
BioRad Goat Anti-Mouse IgG (H + L)- #1706516
Thermo Fisher Scientific # A-11001
Thermo Fisher Scientific # A-11008
Thermo Fisher Scientific # A-11005
Thermo Fisher Scientific # A-11012
Sigma-Aldrich T8660
Thermo Fisher Scientific # 62248
Invitrogen H3570

Validation

Web page from the antibodies providers is linked below, links are ordered according to "Antibodies used" annotation.
https://www.abcam.com/en-us/products/primary-antibodies/coup-tf1-antibody-epr10841-ab181137?srsltid=AfmBOor3qCEHg3OhTSL7UG8SCKelCoN6CP0HOqW-Dg1ZzRXfl0E_knqa
https://www.sigmaaldrich.com/GB/en/product/mm/abe1425?srsltid=AfmBOoo6bFLVXJurya56jee22g6B6P-AWMBKhIaDqcTzZyzazunmu6gv
https://resources.rndsystems.com/pdfs/datasheets/pp-h8132-00.pdf
https://www.cellsignal.com/products/primary-antibodies/coup-tfi-d4h2-rabbit-mab/6364?srsltid=AfmBOoruE4CjiHDd4ev2DAPSeDyYU9PENKAMN5yztnVLbpByzWo0Q3y9
https://www.ptglab.com/products/NR2F1-Antibody-24573-1-AP.htm?srsltid=AfmBOoram_9nCtMAQvo5skQyFdwuPYH_k2_r0QVeRkLLtkTCPUvk3Wwm
https://www.thermofisher.com/antibody/product/NR2F1-Antibody-Polyclonal/PA5-30190
https://www.scbt.com/p/gapdh-antibody-0411?gad_source=1&gclid=CjwKCAiA3ZC6BhBaEiwAeqfvyu63c-nB1e-Q0hQoWbetBYpgX6jKuNJX-J6MpQutkWhM3zk0DjyUMBoC6YcQAvD_BwE
https://www.scbt.com/p/gfp-antibody-c-2?gad_source=1&gclid=CjwKCAiA3ZC6BhBaEiwAeqfvygAJIGTFHtR0H6X7tXpfX8_N0Gc-iSqP0KRYox5knfVgrHK4yPIqnBoCjI0QAvD_BwE
https://www.thermofisher.com/antibody/product/GFP-Antibody-Polyclonal/A-11122
https://www.thermofisher.com/antibody/product/Fibrillarin-Antibody-Polyclonal/PA5-143604
https://www.thermofisher.com/antibody/product/Fibrillarin-Antibody-clone-38F3-Monoclonal/MA3-16771
https://www.thermofisher.com/antibody/product/NPM1-Antibody-clone-FC-61991-Monoclonal/32-5200
https://www.rndsystems.com/products/human-mouse-npm1-antibody_af5205
https://www.sigmaaldrich.com/GB/en/product/mm/ab9354?utm_source=google&utm_medium=cpc&utm_id=21480163361&utm_campaign=%7Bcampaignname%7D&utm_content=167683289209&utm_term=anti-beta+iii+tubulin+antibody&gclid=CjwKCAiA3ZC6BhBaEiwAeqfvyg6fjFSAqwKu1dBG66uoHRDrZkOhxi6J5lJFpcUUkVz2E8DgOGTQvhoCvCcQAvD_BwE

Eukaryotic cell lines

Policy information about cell lines and Sex and Gender in Research

Cell line source(s)

Human: WT hiPSC line T12- DPEDi001-A (male)
Human: WT hiPSC line PGP1 (male)
Human: NR2F1-/- hiPSC line PGP1- C1 (male)
Human: NR2F1+/- hiPSC line PGP1- F3 (male)
Human: HEK293 cell line (female)
Human: HeLa cells (female)

Authentication

All cell lines were purchased from registered provides, in addition to that (with the exeption for HeLa cells), have been karyotyped

Mycoplasma contamination

All cells were routinely checked for mycoplasma and always resulted negative for the tests.

Commonly misidentified lines
(See ICLAC register)

N.A.

Palaeontology and Archaeology

Specimen provenance

N.A.

Specimen deposition

N.A.

Dating methods

N.A.

☐ Tick this box to confirm that the raw and calibrated dates are available in the paper or in Supplementary Information.

Ethics oversight

Identify the organization(s) that approved or provided guidance on the study protocol, OR state that no ethical approval or guidance was required and explain why not.

Note that full information on the approval of the study protocol must also be provided in the manuscript.

Animals and other research organisms

Policy information about studies involving animals; ARRIVE guidelines recommended for reporting animal research, and Sex and Gender in Research

Laboratory animals	COUP-TFInull mouse line were produced in 129S2/SvPas background, the age of each animal used in the study was reported on each experiment.
Wild animals	No wild animals were used in the study.
Reporting on sex	Both males and females mice were used in the study, but the sex was not an exclusion parameter.
Field-collected samples	This study did not involve samples collected in the field.
Ethics oversight	All mouse experiments were conducted in accordance with relevant national and international guidelines and regulations (European Union rules; 2010/63/UE) and have been approved by the local ethical committee in France (CIEPAL NCE/2024-976).

Note that full information on the approval of the study protocol must also be provided in the manuscript.

Clinical data

Policy information about clinical studies

All manuscripts should comply with the ICMJE guidelines for publication of clinical research and a completed CONSORT checklist must be included with all submissions.

Clinical trial registration	N.A.
Study protocol	N.A.
Data collection	N.A.
Outcomes	N.A.

Dual use research of concern

Policy information about dual use research of concern

Hazards

Could the accidental, deliberate or reckless misuse of agents or technologies generated in the work, or the application of information presented in the manuscript, pose a threat to:

No	Yes	
☒	☐	Public health
☒	☐	National security
☒	☐	Crops and/or livestock
☒	☐	Ecosystems
☒	☐	Any other significant area

Experiments of concern

Does the work involve any of these experiments of concern:

No	Yes	
☒	☐	Demonstrate how to render a vaccine ineffective
☒	☐	Confer resistance to therapeutically useful antibiotics or antiviral agents
☒	☐	Enhance the virulence of a pathogen or render a nonpathogen virulent
☒	☐	Increase transmissibility of a pathogen
☒	☐	Alter the host range of a pathogen
☒	☐	Enable evasion of diagnostic/detection modalities
☒	☐	Enable the weaponization of a biological agent or toxin
☒	☐	Any other potentially harmful combination of experiments and agents

Plants

Seed stocks	N.A.
Novel plant genotypes	N.A.
Authentication	N.A.

ChIP-seq

Data deposition

☐ Confirm that both raw and final processed data have been deposited in a public database such as GEO.

☐ Confirm that you have deposited or provided access to graph files (e.g. BED files) for the called peaks.

Data access links<br>*May remain private before publication.*	N.A.
Files in database submission	N.A.
Genome browser session<br>(e.g. UCSC)	N.A.

Methodology

Replicates	N.A.
Sequencing depth	N.A.
Antibodies	N.A.
Peak calling parameters	N.A.
Data quality	N.A.
Software	N.A.

Flow Cytometry

Plots

Confirm that:

☒ The axis labels state the marker and fluorochrome used (e.g. CD4-FITC).

☒ The axis scales are clearly visible. Include numbers along axes only for bottom left plot of group (a 'group' is an analysis of identical markers).

☒ All plots are contour plots with outliers or pseudocolor plots.

☒ A numerical value for number of cells or percentage (with statistics) is provided.

Methodology

Sample preparation	HEK293 cells were dissociated into a single cell suspension by using trypsin-EDTA and transferred into low-bind Eppendorf tubes, washed twice with ice-cold PBS, and fixed with 70% ethanol while mixing on a vortex, and stored at -20°C. 200,000 cells were then stained in suspension, a short centrifugation (5 min at 170 g) was used for the washing steps with 1% serum PBS.
Instrument	BD LSRFortessa system, BD biosciences, https://www.bdbiosciences.com/content/dam/bdb/marketing-documents/bd_lsrfortessa_brochure.pdf
Software	FACSDiva; FlowJo software (Becton Dickinson).

Cell population abundance	For each staining, 10,000 total events (excluding debris) from two independent batches (20,000 cells in total) were measured at the same voltage.
Gating strategy	Viable cells were first selected based on forward and side scatter. A positive gate was then determined for each NR2F1 primary antibody relative to HEK293 cells stained only with the respective secondary antibody. Where positive cells showed more than one log separation, the second gate was added.

☒ Tick this box to confirm that a figure exemplifying the gating strategy is provided in the Supplementary Information.

Magnetic resonance imaging

Experimental design

Design type	N.A.
Design specifications	N.A.
Behavioral performance measures	N.A.

Acquisition

Imaging type(s)	N.A.
Field strength	N.A.
Sequence & imaging parameters	N.A.
Area of acquisition	N.A.

Diffusion MRI ☐ Used ☐ Not used

Preprocessing

Preprocessing software	N.A.
Normalization	N.A.
Normalization template	N.A.
Noise and artifact removal	N.A.
Volume censoring	N.A.

Statistical modeling & inference

Model type and settings	N.A.
Effect(s) tested	N.A.

Specify type of analysis: ☐ Whole brain ☐ ROI-based ☐ Both

Statistic type for inference (See Eklund et al. 2016)	N.A.
Correction	N.A.

Models & analysis

n/a	Involved in the study
☒	☐ Functional and/or effective connectivity
☒	☐ Graph analysis
☒	☐ Multivariate modeling or predictive analysis
